# The specificity trap in routine tumor biomarker interpretation: Advancing the 3PM-based clinical decision framework to benefits of patients

**DOI:** 10.1007/s13167-026-00466-3

**Published:** 2026-08-27

**Authors:** Peter Kubatka, Ludmila Filkova, Iva Slaninova, Tomas Kazda, Karel Smejkal, Dietrich Büsselberg, Olga Golubnitschaja

**Affiliations:** 1 Department of Natural Drugs, Faculty of Pharmacy, Masaryk University, Brno, 625 00 Czech Republic; 2 European Association for Predictive, Preventive and Personalized Medicine (EPMA), Brussels, 1160 Belgium; 3 Department of Biology, Faculty of Medicine, Masaryk University, Brno, 625 00 Czech Republic; 4 Department of Radiation Oncology and Research Centre for Applied Molecular Oncology, Masaryk Memorial Cancer Institute, Brno, 656 53,, Czech Republic; 5 Department of Physiology and Biophysics, Weill Cornell Medicine in Qatar, Qatar Foundation, Education City, Doha, 24144 Qatar; 6 Predictive, Preventive and Personalized (3P) Medicine, Department of Radiation Oncology, University Hospital Bonn, Rheinische Friedrich-Wilhelms-Universität Bonn, 53127 Bonn, Germany

**Keywords:** Routine tumor biomarkers, Predictive preventive personalized medicine (3PM), Cancer biomarker interpretation, Tumor-marker specificity, Longitudinal biomarker monitoring, Clinical decision-making, Systemic effects, Holistic approach, Multilevel diagnostics, Individualized patient profiles, Phenotyping, Mitochondria, AI-based multi-professional data interpretation

## Abstract

Routine tumor biomarkers remain among the most accessible tools in oncology. Yet, their clinical value is frequently weakened by a persistent specificity trap: abnormal results are often treated as cancer-specific signals, whereas normal results are sometimes used to reassure against malignancy. This critical narrative review develops a predictive, preventive, and personalized medicine (3PM)-guided clinical decision framework for interpreting routinely used tumor biomarkers. Evidence was mapped across six domains: validated indication, tumor type and disease phase, baseline marker status, longitudinal trajectory, false-positive and false-negative vulnerability, and analytical or pre-analytical limitations.

The review covers classical circulating epithelial markers, organ-associated markers, neuroendocrine and neural-lineage markers, nonspecific markers of burden or proliferation, tissue-based therapeutic targets, immune-context biomarkers, and less standardized markers with nomenclature-sensitive interpretation. Across these groups, the central synthesis is that clinical usefulness rarely derives from an isolated threshold. It emerges when marker dynamics are interpreted in relation to pre-test probability, comorbidities, renal and liver function, inflammation, medication effects, assay platform, imaging, pathology, symptoms, and actionability. The proposed 3PM-guided workflow shifts routine biomarker use from reactive testing and diagnostic escalation toward context-corrected interpretation, prevention of low-value investigations, and personalized follow-up or treatment reassessment. Routine tumor biomarkers should therefore be reported and interpreted as conditional, trajectory-dependent clinical signals, not as stand-alone evidence for or against cancers.

Finally, in primary care for protecting individuals against health-to-disease transition as well as for individualized rehabilitation programs in secondary care, a 3PM-guided holistic approach is strongly recommended utilizing multi-level diagnostics. This approach is based on comprehensive individualized patient profiles including phenotyping and biomarker panels essentially reflecting systemic effects. To this end, a non-invasive tear fluid test utilizing mitochondria as vital biosensors and AI-based multi-professional data interpretation has been established, followed by the cost-effective targeted disease prevention and individualized rehabilitation programs in primary and secondary care respectively.

**Graphical Abstract:**

**Figure:**
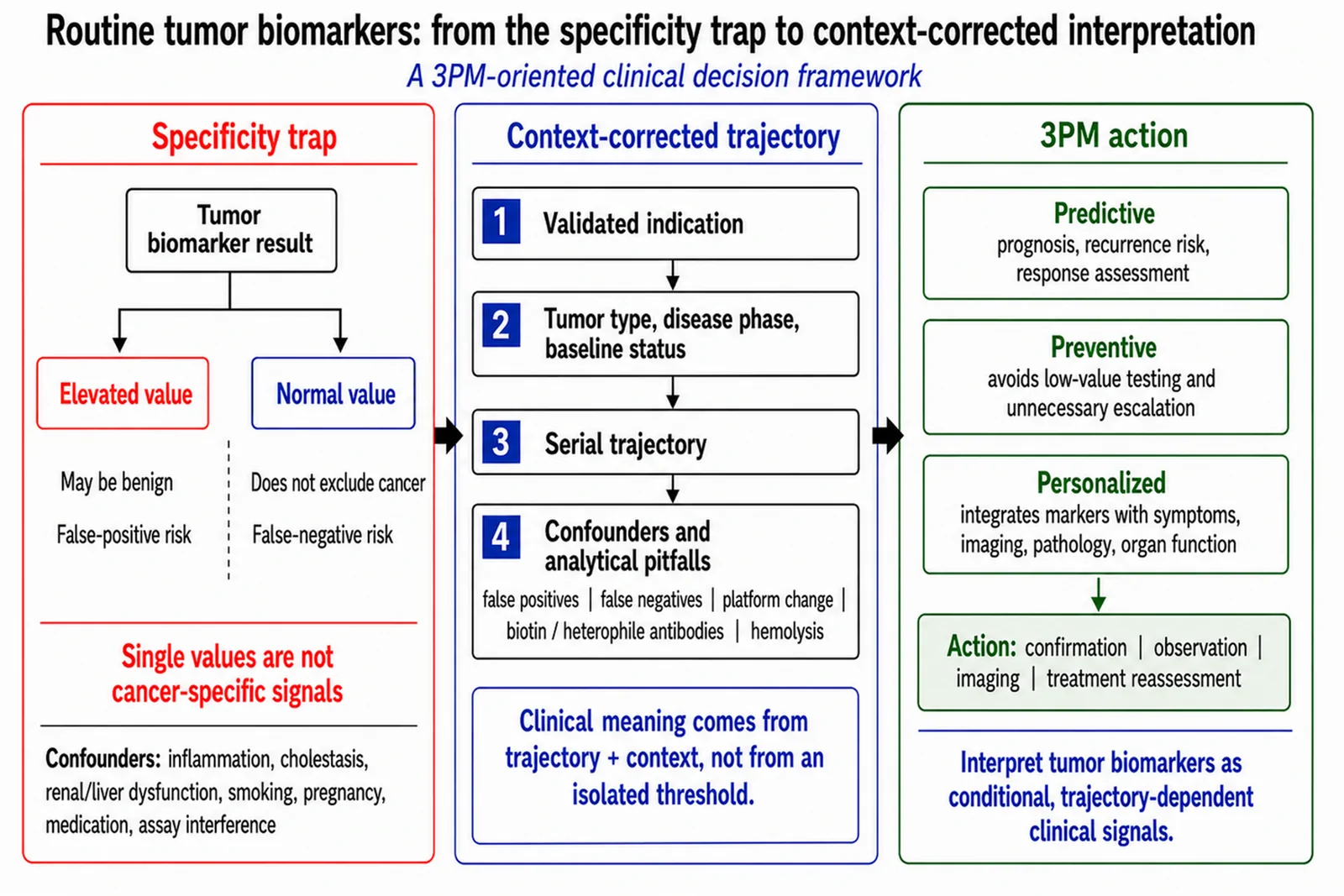

## Introduction: the specificity trap in routine tumor marker interpretation

### Routine tumor biomarkers in clinical practice

Routine tumor biomarkers remain among the most accessible laboratory tools used across oncology and several non-oncological specialties. Serum markers such as carcinoembryonic antigen (CEA), cancer antigen 125 (CA-125), carbohydrate antigen 19 − 9 (CA19-9), cancer antigen 15 − 3 (CA15-3), prostate-specific antigen (PSA), alpha-fetoprotein (AFP), calcitonin, chromogranin A, neuron-specific enolase (NSE), lactate dehydrogenase (LDH), and β2-microglobulin are routinely requested in oncology, internal medicine, surgery, gynecology, urology, endocrinology and, in many healthcare systems, primary care [1–3]. Their clinical appeal is understandable: they are minimally invasive, repeatable, relatively inexpensive, and seemingly objective indicators of malignant disease burden, treatment response, recurrence risk, or disease progression [1, 4].

However, the same accessibility that makes these biomarkers clinically attractive also makes them vulnerable to misuse. Most routine tumor markers are not tumor-specific, organ-specific, or sufficiently sensitive for early cancer detection when used outside validated clinical contexts [2, 5]. Their concentrations may increase in benign inflammatory, hepatic, renal, endocrine, gynecologic, pulmonary, or physiological conditions, while some malignancies may remain marker-negative because of low tumor burden, early-stage disease, non-secretory phenotype, biological heterogeneity, or inappropriate marker selection [2, 3]. Thus, the presence of an abnormal result does not necessarily indicate malignancy, and a normal result does not reliably exclude it.

This creates a central paradox in routine tumor marker interpretation. Tumor biomarkers are clinically useful when they are ordered for a defined indication, interpreted against the correct tumor type and disease phase, and followed longitudinally using a consistent analytical platform [1, 4]. Yet in routine practice, they are often interpreted as if they were cancer-specific signals, particularly when requested as broad diagnostic panels or as screening tests in patients without a clear oncological context [6]. Despite their widespread use, routine tumor biomarkers are frequently interpreted outside validated clinical contexts, creating a clinically relevant gap between laboratory availability and decision-making validity.

The consequence is not merely academic. Overinterpretation of mildly elevated tumor markers may trigger unnecessary imaging, invasive procedures, patient anxiety, and diagnostic escalation. In contrast, overreliance on normal marker values may delay appropriate diagnostic work-up in marker-negative malignancies [2, 6]. The clinical value of these biomarkers, therefore, depends less on the isolated numerical result and more on whether the result is interpreted in relation to indication, baseline value, serial dynamics, tumor biology, comorbidities, organ function, analytical limitations, and clinical actionability. This context-dependent interpretation is consistent with the 3PM paradigm promoted by the European Association for Predictive, Preventive, and Personalized Medicine (EPMA), which emphasizes a shift from reactive medical services toward predictive, preventive, and personalized decision-making [7].

### Aim, working hypothesis, clinical rationale, and novelty of the review

This review does not aim to provide another encyclopedic summary of routinely used tumor markers. Several clinically oriented reviews and guidelines have already described their established roles in diagnosis, prognosis, treatment monitoring, and post-treatment surveillance [1, 8, 9]. The unresolved clinical problem is different: these biomarkers are often available and frequently requested, but their interpretation is not always aligned with validated indications, analytical limitations, or patient-specific context [5, 10].

This critical narrative review aims to develop a clinically applicable interpretation framework for selected biomarkers encountered in clinical oncology, with a primary focus on routinely used circulating tumor markers. CD30, CD38, and TIM-3 are included only as boundary examples of tissue-based therapeutic-target and immune-context biomarkers, whereas TATI/SPINK1/PSTI and TPA are included as nomenclature-sensitive, less standardized comparators. Their inclusion is intended to clarify the limits of transferring interpretive principles across biomarker classes rather than to imply routine use, analytical equivalence, or comparable clinical maturity. We focus on six clinically decisive dimensions: validated indication, tumor type and disease phase, baseline marker status, longitudinal trajectory, false-positive and false-negative vulnerability, and analytical or pre-analytical limitations. This approach is intended to shift interpretation from isolated threshold-based reading toward context-corrected, longitudinal, and clinically actionable assessment.

The novelty of the review lies in integrating routine tumor marker interpretation into a predictive, preventive, and personalized medicine framework. Predictive value in this context is addressed by assessing whether a biomarker can inform prognosis, recurrence risk, treatment response, or disease progression. Preventive value is addressed by identifying situations in which misinterpretation may lead to unnecessary imaging, invasive procedures, patient anxiety, or low-value testing. Personalized value is addressed by incorporating baseline marker status, tumor phenotype, disease phase, comorbidities, renal and liver function, medication effects, assay platform, and serial dynamics into clinical decision-making [10, 11].

The working hypothesis underpinning this review is that the clinical value of routine tumor biomarkers is determined less by isolated threshold crossing than by their interpretation within a validated indication, tumor type, and disease phase, individual baseline, serial trajectory, confounding conditions, and analytical context. We further propose that embedding these dimensions in a 3PM-oriented workflow can improve interpretive validity, reduce avoidable diagnostic escalation and false reassurance, and support more personalized follow-up and treatment reassessment.

## Methodological approach and conceptual framework

### Review design and literature search

This review was designed as a critical narrative review supported by structured clinical evidence mapping. It was not intended to function as a conventional catalog of tumor markers, nor as a systematic review or meta-analysis. Its preparation followed the core principles of transparent narrative synthesis, including a clearly defined rationale, structured literature searching, reference-supported statements, and critical appraisal of the included evidence, consistent with the SANRA framework [12]. Evidence mapping was used to organize a broad and heterogeneous literature base according to predefined clinical domains rather than to generate pooled effect estimates; this approach is particularly suitable for identifying patterns, evidence gaps, and differences in clinical applicability across diverse biomarker classes [13, 14].

PubMed/MEDLINE, Scopus, and Web of Science were searched from database inception to July 8, 2026, with the final search update performed on July 8, 2026. Google Scholar was searched on the same date as a supplementary source for additional clinical guidelines, consensus documents, and relevant records not identified in the principal databases. Searches were conducted separately for each predefined biomarker or recognized synonym. Each biomarker term was combined with terms related to clinical interpretation, sensitivity, specificity, false-positive and false-negative results, analytical interference, biological variation, serial monitoring, prognosis, recurrence, treatment response, clinical utility, guidelines, and expert consensus. Database-specific field tags and syntax were applied. Additional records were identified by manually screening the reference lists of key guidelines and clinically oriented reviews. The representative database-specific search framework and dates of the final searches are provided in Supplementary Table S1.

The searches of PubMed/MEDLINE, Scopus, Web of Science, Google Scholar, and manually screened reference lists yielded a final evidence base of 103 unique sources. Records were assessed against the predefined eligibility criteria and evidence-extraction domains, with exclusion of clearly irrelevant publications, non-human exploratory biomarker-discovery studies, conference-only records, and publications without clinically extractable information. Because record-level counts for initial retrieval, database-level deduplication, title and abstract screening, and full-text exclusion were not prospectively recorded, a PRISMA-style numerical selection flow could not be reconstructed. The final evidence base included clinical practice guidelines, expert consensus documents, laboratory medicine recommendations, systematic reviews, meta-analyses, clinically oriented reviews, and selected original human studies addressing biomarker indications, interpretation, false-positive or false-negative mechanisms, analytical limitations, serial monitoring, or clinical actionability.

Initial title and abstract screening was performed independently by two authors with biomedical and oncological expertise. Potentially relevant full texts were then reviewed against the predefined eligibility criteria and extraction domains. Disagreements were resolved by discussion and, when necessary, by consultation with a senior author with expertise in predictive, preventive, and personalized medicine. Because the aim was clinical interpretation rather than quantitative estimation of diagnostic accuracy, priority was given to clinical practice guidelines, expert consensus statements, and laboratory-medicine recommendations defining validated biomarker indications [4], followed by systematic reviews, meta-analyses, and clinically oriented reviews [1, 3], and original studies providing evidence on diagnostic performance, benign causes of elevation, non-secretory disease, analytical limitations, or longitudinal clinical utility.

### Biomarker selection and scope

The biomarker panel was defined a priori to capture the principal classes of markers encountered in clinical oncology and related specialties. Selection was based on established or historically relevant clinical use, availability in routine or specialized laboratory practice, representation in clinical guidelines, and the ability of individual markers to illustrate recurrent interpretive problems, including limited specificity, non-malignant elevation, non-secretory disease, and assay-dependent variability [1, 4].

The review includes CEA, CA-125, CA19-9, CA-72-4, CA15-3, CYFRA 21 − 1, SCC antigen, HE4, PSA, AFP, calcitonin, thyroglobulin, β-hCG, chromogranin A, 5-HIAA, NSE, S100/S100B, LDH, thymidine kinase/TK1, ferritin, and β2-microglobulin. CD30, CD38, and TIM-3 complemented these circulating biomarkers to distinguish classical serum tumor markers from tissue-based, immunophenotypic, therapeutic-target, and immune-context biomarkers. Their inclusion was intended to clarify differences in the purpose and actionability of biomarkers rather than to imply analytical or clinical equivalence.

Several commonly used, specialized, composite, or emerging biomarker tests were deliberately not included as separate entries. CA27.29 was considered within the same MUC1-associated breast cancer marker logic as CA15-3 rather than treated as a distinct interpretive category. ProGRP, PIVKA-II/DCP, PHI/4Kscore, OVA1/ROMA, and ctDNA-based assays were not excluded because they lack clinical relevance, but because they represent more disease-specific algorithms, composite risk tools, specialized hepatocellular or neuroendocrine applications, prostate-risk stratification tools, adnexal-mass algorithms, or molecular/liquid-biopsy decision pathways that would require separate evidence maps. The aim of this review was therefore not to provide a complete catalog of all available tumor biomarkers, but to analyze selected routine, widely encountered, and interpretation-prone markers whose results are often misread as cancer-specific signals. This scope is consistent with the National Cancer Institute statement that lists of tumor-marker tests in common use are not comprehensive and that new tumor markers frequently become available [15].

TATI/SPINK1/PSTI and tissue polypeptide antigen (TPA) were treated as separate biomarkers. TATI/SPINK1/PSTI represents a trypsin-inhibitory protein with reported diagnostic and prognostic associations in several malignancies [16], whereas TPA comprises circulating cytokeratin fragments and belongs to a distinct group of epithelial cell-turnover markers [17]. No biomarker was included on the assumption that it was sufficiently accurate for stand-alone cancer detection.

### Evidence extraction domains

A predefined extraction framework was applied to each biomarker to improve consistency across biologically and clinically heterogeneous marker classes. Each biomarker was mapped according to principal cancer associations, intended clinical use, false-positive vulnerability, false-negative or non-secretory scenarios, analytical and pre-analytical limitations, serial monitoring value, and decision-making actionability.

Clinical roles were recorded separately for screening, diagnosis, prognosis, treatment monitoring, and recurrence surveillance because evidence supporting one application, such as monitoring established disease, cannot be extrapolated automatically to screening or primary diagnosis [4, 18]. For serially measured biomarkers, particular attention was given to baseline marker status, direction and magnitude of change, biological variation, analytical imprecision, and concordance with clinical or imaging findings. Previous measurements from the same patient may provide a more informative comparator than a population reference limit, especially when changes remain within expected analytical and intra-individual variation [19]. Evidence for longitudinal use was therefore distinguished from evidence based solely on cross-sectional diagnostic performance, in line with recommendations that biomarker kinetics, disease-status discrimination, and the clinical consequences of marker-guided intervention should be evaluated separately [20].

Clinical actionability was defined as the extent to which a biomarker result could reasonably alter diagnostic work-up, imaging, surveillance intensity, prognostic stratification, treatment selection, or response assessment. Interpretation also considered whether the underlying assay had adequate analytical validation, whether the proposed clinical use had been independently validated, and whether biomarker-guided decisions were supported by evidence of clinical utility [21]. This structure allowed markers with strong disease-specific applications to be distinguished from adjunctive, burden-related, poorly standardized, or predominantly investigational biomarkers.

### Eligibility criteria

Publications were eligible when they provided clinically relevant human evidence on the indication, performance, interpretation, or analytical limitations of at least one predefined biomarker. Eligible sources comprised clinical practice guidelines and expert consensus statements; laboratory-medicine recommendations; systematic reviews and meta-analyses; clinically oriented reviews; and observational or interventional human studies. Publications were included when they addressed at least one predefined domain: screening or diagnostic performance, prognostic value, treatment monitoring, recurrence surveillance, false-positive elevation, false-negative or non-secretory states, biological variation, analytical interference, assay-related limitations, or serial biomarker kinetics. Only English-language full-text publications identified from database inception through July 8, 2026, were considered.

Publications were excluded if they did not address the predefined biomarker panel; lacked a clinically interpretable human context; focused exclusively on exploratory biomarker discovery without routine or emerging clinical applicability; or provided insufficient methodological or outcome information for structured evidence mapping. Conference abstracts, letters, editorials, and opinion-based commentaries without extractable clinical evidence were excluded. Isolated case reports were generally excluded unless they documented a clinically important analytical interference or an unusual false-positive or false-negative mechanism. Superseded guidelines were replaced by their most recent versions unless an earlier document was required to clarify the historical development of a recommendation.

### Methodological limitations

This review should not be interpreted as a systematic review, scoping review, or meta-analysis. No pooled estimates of sensitivity, specificity, predictive value, hazard ratios, or diagnostic odds ratios were calculated, and no formal risk-of-bias scoring was applied across all included studies. The purpose was instead to provide a critical narrative synthesis supported by structured evidence mapping across clinically relevant interpretive domains. The resulting framework is therefore intended as a practical interpretation tool for routine tumor-marker use, not as a replacement for marker-specific clinical guidelines or disease-specific diagnostic algorithms.

Several methodological limitations should be acknowledged. First, the literature base was broad and heterogeneous, including biomarkers with substantially different levels of analytical validation, clinical validation, and clinical utility. Second, only English-language full-text publications were considered, potentially excluding relevant regional or non-English data. Third, because the review focused on routinely used and clinically illustrative biomarkers, it does not provide an exhaustive catalog of all available molecular, genomic, imaging, or liquid-biopsy biomarkers. Fourth, actionability categories were assigned as pragmatic marker–indication judgments rather than as a formally validated scoring system. Finally, the clinical usefulness of a tumor marker may change with new assays, evolving treatment pathways, improved imaging, molecular diagnostics, and updated disease-specific guidelines; the proposed framework should therefore be interpreted as a decision-support structure rather than a fixed classification.

## General principles of tumor biomarker interpretation

### Sensitivity, specificity, and predictive value

The biological link between a marker and malignant disease is not, by itself, evidence of diagnostic usefulness. Many circulating tumor markers originate from proteins expressed by both malignant and non-malignant tissues, or reflect processes such as epithelial turnover, inflammation, and tissue injury. A marker may therefore correlate with tumor burden in patients with established cancer and still perform poorly when used to determine whether cancer is present [1, 9].

Clinical sensitivity describes the proportion of patients with the target disease who have a positive test result, whereas clinical specificity refers to the proportion of individuals without that disease who are correctly classified as negative [22, 23]. These measures characterize test performance in a defined population, but they do not directly answer the clinician’s central question: how likely is cancer in this particular patient after an abnormal result? The positive predictive value expresses the probability that the disease is present when the test is positive, whereas the negative predictive value estimates the probability that the disease is absent when the test is negative [22, 23].

Unlike sensitivity and specificity, predictive values depend strongly on disease prevalence and pre-test probability. This distinction is particularly important when tumor markers are applied outside oncology. In an asymptomatic population, where cancer prevalence is low, even a relatively specific marker may produce more false-positive than true-positive results [24]. CA19-9 illustrates this problem well. Although it may contribute to the assessment of patients with suspected or established pancreaticobiliary malignancy, its positive predictive value is inadequate for population screening [25].

The required performance also varies depending on the clinical purpose. Screening demands high sensitivity for early, potentially curable disease, but also very high specificity, because even a low false-positive rate can lead to a large number of unnecessary investigations. Diagnostic use involves a clinically selected population and a different pre-test probability. Once malignancy has been confirmed, the same marker may be useful for estimating tumor burden, monitoring treatment, or supporting the detection of recurrence, even if it was not sufficiently sensitive for early diagnosis [1].

These clinical roles should not be treated as interchangeable. Evidence that a marker is associated with prognosis does not establish diagnostic accuracy. Likewise, earlier biochemical detection of recurrence is not necessarily beneficial unless it leads to an intervention that improves a meaningful clinical outcome, as illustrated by recommendations against routine serum tumor-marker surveillance after primary breast cancer treatment [26, 27]. Sensitivity, specificity, and predictive values must therefore be interpreted for a defined tumor type, disease stage, patient population, decision threshold, and intended use [28].

The practical meaning of an identical laboratory value can consequently differ greatly between an asymptomatic individual, a patient undergoing investigation for a suspected malignancy, and a patient being monitored after cancer treatment. Ignoring these differences is one of the main reasons why tumor markers continue to generate misleading results and low-value diagnostic procedures in routine practice [6].

### Single result versus longitudinal dynamics

A single tumor marker result is only a snapshot. It reflects not only tumor secretion but also the patient’s usual baseline, biological fluctuation, organ function, concurrent disease, and analytical variation. A mildly elevated value may therefore indicate malignancy, a benign transient change, a stable individual baseline, or an analytical artifact. Conversely, a result that remains within the population reference interval may still represent a meaningful increase for a particular patient [19].

Serial measurements are often more informative, provided that the marker was relevant at baseline and subsequent samples were collected under comparable conditions. Useful longitudinal features include the pretreatment value, the postoperative or post-treatment nadir, the magnitude and direction of change, a sustained rise across consecutive samples, doubling time, velocity, and percentage change. None of these should be interpreted in isolation from treatment timing, symptoms, imaging, and the expected biological behavior of the tumor [29].

A persistent rise is generally more concerning than a marginally abnormal result. A fall after treatment may support a response, but it cannot independently establish a response without clinical and radiological assessment. The same caution applies to an increase: marker progression is not automatically equivalent to radiological or clinical progression [1].

The distinction between a true biological change and ordinary variation is central to longitudinal interpretation. Both analytical imprecision and within-person biological variation contribute to differences between consecutive measurements. In clinically stable patients with ovarian cancer, these components materially influenced serial CA-125 values, suggesting that previous results from the same patient may be more informative than the population reference limit alone [19]. Biological variation has also been described for CA19-9, CEA, and AFP, supporting the use of marker-specific reference change values rather than a universal percentage threshold [29, 30].

Longitudinal algorithms can improve discrimination in selected settings. Serial CA-125 assessment based on changes over time identified ovarian cancer earlier than a conventional single-threshold strategy in a screening cohort [31]. In breast cancer surveillance, changes in CA15-3 and CEA over time were more informative than isolated concentrations in a retrospective analysis [32]. These findings support a dynamic interpretation, but they should not be automatically extended to other patient populations, assays, or clinical purposes.

In practice, serial measurements should be performed under comparable pre-analytical conditions and, whenever possible, in the same laboratory using the same analytical method, as results obtained with different assay platforms may not be directly interchangeable [33]. A change is most credible when it exceeds the expected contribution of analytical imprecision and within-person biological variation and remains consistent with the broader clinical context [19, 30]. When the marker trajectory conflicts with imaging findings, symptoms, or the observed treatment response, the discrepancy should be investigated rather than used alone as an immediate basis for changing management [34].

### False-positive, false-negative, and analytical pitfalls

Most conventional tumor markers are not produced exclusively by malignant tissue. False-positive results are therefore an inherent limitation rather than an occasional exception. Inflammation and infection may increase epithelial turnover or alter marker release, whereas liver dysfunction and cholestasis can affect biomarker synthesis, metabolism, and biliary clearance. These processes may produce clinically relevant increases in CEA, CA19-9, or CA-125 without corresponding tumor progression. Renal dysfunction creates a similar problem by reducing the clearance of protein biomarkers, including β2-microglobulin, chromogranin A, HE4, and SCC antigen [1, 35].

Physiological and benign conditions add further complexity. Pregnancy alters AFP and β-hCG concentrations and may influence several carbohydrate antigens, while menstruation, endometriosis, and other benign gynecologic disorders can increase CA-125. PSA may rise in benign prostatic hyperplasia, prostatitis, or after recent prostatic manipulation, and smoking is a recognized cause of higher CEA concentrations. Medication history is equally important: proton pump inhibitors can cause substantial chromogranin A elevation that is unrelated to neuroendocrine tumor activity [36].

These confounding factors are not necessarily stable during follow-up. Renal function, cholestasis, inflammation, smoking exposure, or medication use may vary between measurements, generating an apparent biomarker trajectory that resembles disease progression or treatment response. A rise in a tumor marker should therefore be interpreted alongside contemporaneous renal, hepatic, and inflammatory indices and any relevant change in clinical status or treatment [35].

False-negative results arise through different mechanisms. Early-stage or low-volume tumors may release too little analyte to exceed the selected decision threshold, and some malignancies are biologically non-secretory. Marker expression may also vary between histological subtypes, between primary and metastatic lesions, or during treatment. CA19-9, for example, may remain low in patients who cannot express the relevant antigen because of their Lewis phenotype. Poorly differentiated tumors may likewise lose the production of markers associated with their tissue of origin. A normal marker concentration should therefore not be used to exclude malignancy unless the assay has been validated for that specific diagnostic purpose [1].

Pre-analytical factors may further distort the result before the analytical measurement begins. Hemolysis is particularly important for NSE because erythrocytes and platelets contain enolase, and even limited cellular disruption may produce a falsely elevated concentration. Delayed processing, unsuitable specimen type, prolonged storage, repeated freeze–thaw cycles, and inconsistent collection timing may also influence selected biomarkers. When a result is unexpected or poorly aligned with the clinical picture, specimen quality and handling should be reviewed before the change is attributed to malignancy [37].

Most circulating tumor markers are measured by immunoassays, which remain vulnerable to analytical interference. Heterophile antibodies and human anti-animal antibodies can interact with assay reagents and produce falsely high or falsely low concentrations [38]. In a recent report, heterophile antibody interference caused persistent platform-specific elevation of CA19-9 and led to unnecessary diagnostic investigations; the discrepancy became evident only after analysis on alternative platforms and treatment with a blocking reagent [39].

Biotin is another relevant source of error in assays that use biotin–streptavidin binding. Depending on the assay format, excess circulating biotin may cause either falsely low or falsely high results [40]. Supplement use should therefore be considered when a laboratory result is unexpected or inconsistent with the clinical picture.

At very high analyte concentrations, immunometric assays may also show a high-dose hook effect. Excess antigen prevents the normal formation of antibody–antigen complexes and yields a deceptively low or occasionally negative result. The effect has been reported for markers including PSA and β-hCG, with the true concentration becoming apparent only after sample dilution [41]. A hook effect should be suspected when the measured value is clearly incompatible with the disease severity.

Assay results are also not fully interchangeable between manufacturers. Differences in antibodies, calibration systems, epitope recognition, and traceability can produce clinically important discrepancies. A large external quality-assessment analysis published in 2024 found substantial manufacturer-dependent variation in CA15-3, CA19-9, and CA-125 results, despite generally acceptable precision within individual methods [33]. Changing the analytical platform during follow-up may therefore create an apparent rise or fall that is methodological rather than biological.

**Fig. 1:**
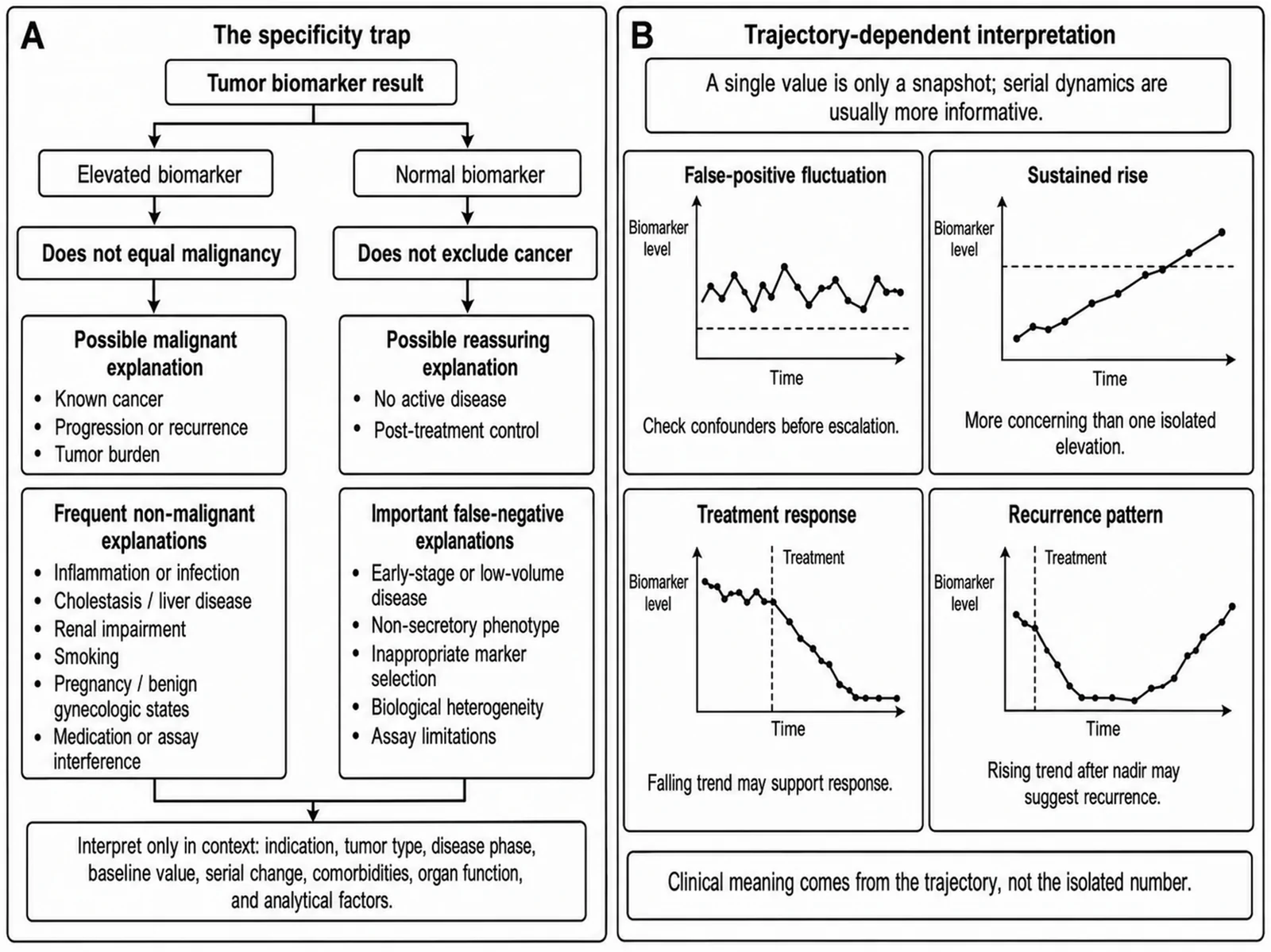
The specificity trap and trajectory-dependent interpretation of tumor biomarkers The figure illustrates why routine tumor biomarkers should not be interpreted as isolated cancer-specific signals. (A) An elevated biomarker value does not necessarily indicate malignancy because non-malignant conditions such as inflammation, cholestasis, liver or renal dysfunction, smoking, pregnancy-related or benign gynecologic states, medication effects, and assay interference may generate false-positive results. Conversely, a normal biomarker value does not exclude cancer, particularly in early-stage, low-volume, non-secretory, biologically heterogeneous, or marker-inappropriate disease. Biomarker interpretation therefore requires integration of the clinical indication, tumor type, disease phase, baseline value, comorbidities, organ function, serial change, and analytical context. (B) Serial trajectories are usually more informative than single measurements. A transient or fluctuating abnormality should prompt assessment of confounders, whereas a sustained rise across consecutive measurements is more clinically concerning. A downward trajectory after treatment may indicate treatment response, whereas a renewed increase after a post-treatment nadir may suggest recurrence or progression. Clinical meaning, therefore, derives from the biomarker pattern over time rather than from an isolated numerical value.

When a result is unexpected, repeating the measurement on the same sample is not always sufficient, because the same interference may recur. Depending on the suspected problem, useful steps include sample dilution, heterophile-blocking procedures, repeat sampling, and measurement with a different assay platform. Close communication between the treating clinician and the laboratory is essential. A result that does not fit the patient should first be questioned, not forced into the presumed disease narrative [42]. These principles are summarized in Fig. 1, which illustrates the specificity trap of isolated tumor-marker values and the added clinical value of trajectory-dependent interpretation.

## Clinically relevant taxonomy of selected tumor biomarkers

A clinically useful taxonomy should do more than group markers by molecular structure. It should indicate what kind of information a marker can reasonably provide, under which conditions that information is valid, and where interpretation is likely to fail. The categories used here are therefore operational rather than absolute. They separate classical circulating markers from organ-associated biomarkers, phenotype-dependent neuroendocrine markers, nonspecific indicators of burden or proliferation, and tissue-based therapeutic targets. This distinction is important because biological association, analytical measurability, and clinical actionability are not equivalent properties [3, 21, 28]. To make this operational taxonomy explicit, Table 1 groups the selected biomarkers by the type of clinical information they can provide, the main logic behind their interpretation, and the principal context in which their use may be misleading.

**Table 1:** Clinically relevant taxonomy of selected tumor biomarkers

Biomarker group	Included markers	Biological/clinical logic	Main interpretive risk
Classical circulating epithelial/glycoprotein markers	CEA; CA-125; CA19-9; CA-72-4; CA15-3; CYFRA 21 − 1; SCC antigen; HE4	Widely available circulating markers are used mainly as adjuncts in epithelial malignancies. Their value is strongest when the tumor type is known, the marker is informative at baseline, and serial changes are interpreted in a clinical context.	Elevation is often non-specific. Inflammation, serosal irritation, cholestasis, smoking, renal or liver dysfunction, and benign epithelial disease may mimic malignancy or progression.
Organ-associated markers with stronger clinical niches	PSA; AFP; β-hCG; calcitonin; thyroglobulin	Markers are more closely linked to a specific organ, lineage, or tumor entity. Their clinical specificity emerges mainly in defined settings, such as germ-cell tumors, medullary thyroid carcinoma, differentiated thyroid carcinoma after thyroidectomy, or risk-adapted prostate assessment.	Organ-associated does not mean cancer-specific. Benign tissue activity, physiological states, organ injury, treatment status, and assay interference can substantially alter interpretation.
Neuroendocrine and neural-lineage markers	Chromogranin A; 5-HIAA; NSE; S100/S100B	Markers reflecting secretory, neuroendocrine, high-grade neuroendocrine, or neural/melanocytic phenotype rather than anatomical origin alone. They are most useful when the tumor is expected to produce the analyte and the marker is elevated at baseline.	A normal result may indicate non-secretory biology rather than the absence of disease. Medication, diet, renal impairment, hemolysis, tissue injury, and non-malignant neurological or systemic conditions may cause misleading elevations.
Nonspecific markers of tumor burden, cell turnover, injury, and proliferation	LDH; TK1; ferritin; β2-microglobulin	Biologically heterogeneous markers that often reflect tumor burden, proliferation, cellular injury, systemic inflammation, immune activation, or impaired clearance. Their strongest role is usually prognostic or longitudinal within a defined disease context.	Very low cancer specificity. Hemolysis, liver or muscle injury, infection, inflammation, iron overload, transfusion history, renal dysfunction, or metabolic disease may dominate the result.
Tissue, therapeutic-target, and immune-context markers	CD30; CD38; TIM-3	Markers are assessed mainly by tissue-based, flow cytometric, or immunochemical methods. They may define cellular phenotype, therapeutic eligibility, minimal residual disease context, immune dysfunction, or resistance biology.	Interpretation depends on the cell population expressing the marker, staining pattern, assay method, prior therapy, and disease context. Reactive immune-cell expression should not be confused with actionable tumor-cell expression.
Nomenclature-sensitive and less standardized markers	TATI/SPINK1/PSTI; TPA	Markers whose interpretation is complicated by terminology, molecular form, or assay definition. TATI/SPINK1/PSTI denotes the same trypsin-inhibitory protein in different contexts, whereas TPA is an assay-defined cytokeratin-associated marker.	Abbreviated names may obscure what was actually measured. Sample type, assay target, molecular form, and terminology must be specified before clinical or literature-based interpretation is attempted.

Table note: The categories are operational rather than absolute. They define the dominant interpretive logic of each marker group, not all possible clinical uses. Actionability categories used throughout the review are defined as follows: **A**, established use in a defined clinical context; **B**, clinically useful adjunct but not a standalone test; **C**, mainly prognostic, burden-related, or longitudinal use; and **D**, investigational, poorly standardized, or highly context-dependent use. Individual markers may move between categories depending on tumor type, disease phase, assay platform, baseline status, and whether the result can change a clinical decision

### Classical circulating epithelial/glycoprotein markers

This group brings together several biologically different analytes that are commonly treated as a single class in routine practice. CEA is an oncofetal glycoprotein; CA-125, CA19-9, CA-72-4, and CA15-3 are carbohydrate or mucin-associated antigens; CYFRA 21 − 1 reflects circulating cytokeratin 19 fragments; SCC antigen belongs to the serpin family; and HE4 is a secreted glycoprotein initially characterized in the epididymis. Their common feature is not a shared molecular origin, but their widespread use as circulating adjuncts in the management of epithelial malignancies [3].

These markers have genuine clinical value, but mostly within narrowly defined settings. CEA is established primarily for prognosis and surveillance in colorectal cancer rather than for population screening. CA15-3 may support monitoring in metastatic breast cancer, whereas CA-125 is most informative in the follow-up of epithelial ovarian cancer and in the assessment of an adnexal mass when interpreted alongside imaging, menopausal status, and clinical findings [43]. HE4 can add information to CA-125-based risk assessment, but its value is also context-dependent and is influenced by factors such as age, smoking, and renal function [3, 44].

CA19-9 is used primarily for pancreaticobiliary malignancies, particularly to assess disease course and treatment response. Its limitations are substantial: cholestasis and benign pancreatobiliary disease can produce marked elevations, while patients with a Lewis-negative phenotype may not express the antigen even in advanced cancer. CA-72-4 has been studied mostly in gastric and mucinous malignancies. Although it may contribute to prognosis or recurrence monitoring in selected patients, its low sensitivity and very low positive predictive value in asymptomatic populations make it unsuitable for general gastric cancer screening [45–47].

CYFRA 21 − 1 and SCC antigen are most commonly discussed in relation to squamous cell malignancies, including lung, head and neck, cervical, and esophageal cancers. Their concentrations tend to increase with disease burden, and they may provide prognostic or monitoring information in selected settings. Neither marker, however, has sufficient organ specificity to identify the primary site or establish malignancy without histological and radiological confirmation [3, 48, 49].

The main interpretive error within this group is to treat an elevated concentration as a disease label. These markers are better understood as conditional signals. Their meaning depends on the suspected tumor, disease stage, baseline secretion, organ function, and serial behavior. In most cases, their strongest role begins after a malignancy has already been established, not before [9].

### Organ-associated markers with stronger clinical niches

PSA, AFP, calcitonin, thyroglobulin, and β-hCG are more closely linked to specific organs, cell lineages, or tumor entities than most classical epithelial markers. This narrower biological association gives them stronger clinical niches, but it does not make them universally cancer-specific. Their usefulness still depends on the question being asked and the patient population in which the measurement is made.

PSA illustrates this distinction particularly well. In patients with established prostate cancer, serial total PSA measurements provide valuable information on treatment response, residual disease, and recurrence. In the prediagnostic setting, however, total PSA is prostate-specific rather than prostate-cancer-specific, because benign prostatic hyperplasia, inflammation, and recent manipulation may increase its concentration. When deciding whether prostate MRI or biopsy is justified, risk assessment can therefore be refined using the free-to-total PSA ratio, PSA density, PSA kinetics, the Prostate Health Index, or the 4Kscore, together with age, prostate volume, previous PSA values, life expectancy, and the overall clinical risk profile [45, 50–52].

AFP and β-hCG occupy a more firmly established position in germ-cell oncology. Together with LDH, AFP and β-hCG contribute to diagnosis, staging, risk classification, treatment monitoring, and post-treatment surveillance in testicular germ-cell tumors [53]. AFP and β-hCG also have established roles in selected ovarian germ-cell tumors. Their pattern also carries histological information: a true AFP elevation is incompatible with pure seminoma and suggests a nonseminomatous component. Even here, context remains essential. AFP may rise in pregnancy, chronic liver disease, and hepatocellular regeneration, while β-hCG can be affected by pregnancy, pituitary secretion, renal dysfunction, and immunoassay interference [45, 54, 55].

In hepatocellular carcinoma, AFP is most informative when interpreted in a population already known to be at risk and combined with imaging. A normal value does not exclude hepatocellular carcinoma, and an elevated value is not diagnostic in a patient with active liver injury. Its clinical contribution lies in risk-adapted surveillance, prognosis, and response assessment rather than in indiscriminate testing [45].

Calcitonin is closely linked to parafollicular C-cell biology and remains the central circulating marker for medullary thyroid carcinoma. Basal concentrations, postoperative nadir, and doubling time can provide information on residual disease and progression. Mild elevations are less straightforward because renal dysfunction, C-cell hyperplasia, smoking, medication, and assay-related factors may contribute. Rare calcitonin-low or calcitonin-negative medullary tumors further show that even a biologically well-matched marker is not infallible [56–58].

Thyroglobulin has a different logic. It is produced by normal as well as differentiated malignant thyroid follicular cells and is therefore poorly suited to distinguishing a benign thyroid nodule from cancer when the thyroid gland remains in place. Its clinical specificity emerges mainly after total thyroidectomy, with or without radioiodine ablation, when persistent or rising thyroglobulin may indicate residual or recurrent differentiated thyroid carcinoma. Anti-thyroglobulin antibodies, thyroid-stimulating hormone status, and assay method must be considered with every measurement [59, 60].

The strength of this category lies in defined clinical niches rather than universal diagnostic performance. These markers can become highly informative when the relevant organ, tumor type, and treatment state are known. Outside that setting, their apparent specificity falls quickly.

### Neuroendocrine and neural-lineage markers

Chromogranin A, 5-HIAA, NSE, and S100/S100B are linked less to a single anatomical site than to a secretory, neuroendocrine, or neural phenotype. Their concentration therefore depends on what the tumor produces, how well differentiated it remains, and how the analyte is processed by the body. A negative result may simply mean that the tumor does not express or release that particular marker.

Chromogranin A is stored in dense-core secretory granules and can be elevated in a broad range of well-differentiated neuroendocrine neoplasms. It may correlate with tumor burden and can support follow-up when clearly elevated at baseline. Its broad expression is also its main weakness. Proton pump inhibitors, atrophic gastritis, renal impairment, cardiovascular disease, and other non-malignant conditions can produce substantial increases. In addition, assays differ in antibody specificity and calibration, limiting the comparability of results across platforms [61, 62].

5-HIAA, the principal metabolite of serotonin, is more phenotype-specific. It is most useful in patients with serotonin-secreting neuroendocrine tumors and carcinoid syndrome, particularly those arising from the midgut. Tumors that do not produce serotonin may remain biochemically silent. Diet, medication, completeness of urine collection, and renal function also influence the result, so pre-test preparation is part of the assay rather than an optional detail [61, 63, 64].

NSE reflects neuroendocrine differentiation and is used mainly as an adjunctive marker in small-cell lung cancer and other high-grade neuroendocrine malignancies. It may carry prognostic information, but its diagnostic specificity is limited. Hemolysis is a major practical problem because erythrocytes and platelets contain enolase and can generate a misleading elevation. The result should therefore be questioned when it is discordant with specimen quality or the clinical course [37, 64, 65].

S100B is most established as a prognostic and monitoring marker in advanced melanoma. Higher concentrations often accompany greater disease burden and poorer outcomes, yet S100B is also released in neurological injury and several non-malignant systemic conditions. It is consequently more useful for risk stratification or longitudinal follow-up in a patient with known melanoma than for detecting melanoma in an unselected population [66–68].

These markers should be viewed as readouts of tumor phenotype rather than universal indicators of neuroendocrine or neural malignancy. Their interpretation begins with a biological question: does this particular tumor have the capacity to produce the measured analyte?

### Nonspecific markers of tumor burden, cell turnover, injury, and proliferation

Lactate dehydrogenase (LDH), thymidine kinase 1 (TK1), ferritin, and β2-microglobulin are biologically heterogeneous markers, but they share an important clinical characteristic: their circulating concentrations frequently reflect tumor burden, cellular proliferation, tissue injury, systemic inflammation, or impaired clearance rather than a cancer-specific signal. Their principal value, therefore, lies in prognostic stratification and longitudinal monitoring within defined disease contexts, while their suitability for population screening or standalone cancer diagnosis is limited.

LDH catalyzes the reversible conversion of pyruvate and lactate and occurs as five isoenzymes composed of different combinations of the A and B subunits. Increased total serum LDH may reflect rapid tumor growth, hypoxia, necrosis, and high cellular turnover, but similar elevations occur in hemolysis, liver or muscle injury, infection, and systemic inflammation. In oncology, LDH is primarily a nonspecific marker of disease burden and adverse prognosis. It contributes to established risk-classification systems in selected malignancies, including metastatic germ-cell tumors, and has prognostic relevance in several hematological and advanced solid cancers [69]. However, LDH is generally prognostic rather than treatment-predictive, and an isolated elevation should not be used to identify the primary tumor site or select therapy. Serial changes are clinically meaningful only when hemolysis, organ injury, and inflammatory conditions have been excluded, and the trajectory is concordant with imaging and the clinical course.

TK1 is a cytosolic enzyme of the pyrimidine salvage pathway whose expression and activity increase during the DNA-synthesis phase of the cell cycle. Circulating TK1 activity or protein concentration may therefore provide information on proliferative activity and has been investigated for prognostic assessment, treatment monitoring, and detection of recurrence, particularly in hematological malignancies and selected solid tumors [70–72]. Nevertheless, available assays measure different molecular properties, including enzymatic activity and TK1 protein concentration, and their results and decision thresholds are not directly interchangeable. TK1 is consequently not established as a general cancer-screening marker or as a standalone diagnostic test. Its most plausible role is as a context-dependent longitudinal marker when the same validated method is used, and changes are interpreted together with established clinical and imaging endpoints.

Ferritin is an intracellular iron-storage protein and a circulating acute-phase reactant. Hyperferritinaemia has been reported in several advanced solid and hematological malignancies and may correlate with systemic inflammation, tumor burden, and adverse prognosis [73, 74]. Its diagnostic specificity is, however, very low. Infection, inflammatory disease, liver injury, iron overload, repeated transfusions, renal dysfunction, and metabolic disorders may all increase serum ferritin independently of malignancy. Ferritin should therefore not be interpreted as evidence of cancer in isolation. When used in an oncological context, its concentration should be assessed alongside inflammatory markers, liver function, iron indices, treatment history, and the broader disease trajectory.

β2-microglobulin is the light-chain component of major histocompatibility complex class I molecules and is released during normal and pathological cellular turnover. Serum β2-microglobulin has established prognostic relevance in plasma-cell and lymphoid malignancies and is incorporated into risk-stratification systems for multiple myeloma. Elevated concentrations have also been associated with greater disease burden and inferior outcomes in chronic lymphocytic leukemia and aggressive B-cell lymphomas [75–77]. However, β2-microglobulin is cleared predominantly by the kidneys and may increase substantially in renal impairment or systemic inflammation. Renal function is therefore an obligatory component of its interpretation. Circulating β2-microglobulin should also be distinguished from tumor-specific B2M gene alterations, which represent a separate tissue or molecular biomarker of immune escape and cannot be inferred from the serum concentration.

Within the actionability framework defined in Table 1, most markers in this group fall predominantly into category C and are used mainly for prognosis, burden assessment, or longitudinal monitoring. Their interpretation should consider baseline values, organ function, inflammation, and changes in disease status.

### Boundary category: tissue-based therapeutic targets and immune-context biomarkers

These biomarkers are included as boundary examples to prevent inappropriate conceptual transfer from circulating tumor markers to tissue-based therapeutic targets and immune-context markers. CD30, CD38, and TIM-3 differ fundamentally from conventional circulating tumor markers. Their main value lies in defining cellular phenotype, therapeutic eligibility, immune context, or treatment resistance rather than in detecting malignancy from an isolated serum concentration. They may be assessed by immunohistochemistry, flow cytometry, multiparameter immunophenotyping, or, for TIM-3, as membrane-bound, exosomal, or soluble forms. Because these molecules are also expressed by normal or reactive immune cells, interpretation must identify the relevant cellular compartment. The key question is not simply whether a marker is present, but which cells express it, how strongly and homogeneously, and whether this expression supports a validated clinical intervention.

CD30 is a member of the tumor necrosis factor receptor superfamily encoded by *TNFRSF8*. It is strongly expressed by neoplastic cells in classical Hodgkin lymphoma and anaplastic large-cell lymphoma, with variable expression in other lymphoid malignancies. However, activated lymphocytes and other immune cells may also express CD30 during infection, inflammation, or autoimmune disease. This is particularly important in classical Hodgkin lymphoma, where neoplastic Hodgkin and Reed–Sternberg cells may constitute only a small fraction of a predominantly reactive cellular background. CD30 positivity should therefore be interpreted together with morphology, complete immunophenotype, and clinical context [78].

The principal actionability of CD30 is its role as the target of brentuximab vedotin. CD30 expression supports CD30-directed treatment in specific settings, particularly classical Hodgkin lymphoma and systemic anaplastic large-cell lymphoma, but cannot independently establish either diagnosis. In anaplastic large-cell lymphoma, ALK status remains essential for classification and prognosis; however, CD30 rather than ALK is the direct therapeutic target, and treatment activity is not restricted to ALK-positive disease [78, 79]. CD30 expression may also decline or be lost under therapeutic pressure, particularly in anaplastic large-cell lymphoma after brentuximab vedotin-containing therapy [80]. Reassessment at progression may therefore be appropriate when further CD30-directed treatment is considered. Sampling error, limited biopsy material, fixation, staining conditions, and heterogeneous expression remain additional causes of an apparently negative result.

CD38 is a multifunctional transmembrane glycoprotein with receptor and ectoenzymatic functions. It is expressed by plasma cells and several immune-cell populations and is particularly abundant in multiple myeloma. Clinically relevant expression also occurs in chronic lymphocytic leukemia, acute leukemias, selected lymphomas, and immunosuppressive populations within some solid-tumor microenvironments [81, 82]. Its broad physiological distribution means that CD38 positivity alone is neither tumor-specific nor sufficient for disease classification.

The strongest clinical actionability of CD38 is found in plasma-cell disorders. Anti-CD38 monoclonal antibodies, particularly daratumumab and isatuximab, are major components of multiple myeloma treatment, while daratumumab is also used in systemic light-chain amyloidosis. CD38 may additionally contribute to residual-disease assessment within a multiparameter flow-cytometric panel. Interpretation after anti-CD38 therapy is more difficult because antigen downregulation, internalization, or epitope masking may reduce apparent detection; alternative antibody clones and additional plasma-cell markers may therefore be required. Outside established plasma-cell indications, CD38-directed antibodies, cell-based therapies, and theranostic approaches remain largely investigational [83].

TIM-3, also designated CD366 or HAVCR2, is an immune-regulatory receptor expressed on several T-cell, natural killer-cell, dendritic-cell, and myeloid populations, as well as on selected malignant and leukemic stem cells. Its relevance lies mainly in immune-state characterization. Persistent TIM-3 expression, especially with other inhibitory receptors, may identify dysfunctional or exhausted immune-cell populations and has been associated with advanced disease, treatment resistance, and an immunosuppressive tumor microenvironment [84]. TIM-3 also occurs in exosomes and as soluble sTIM-3. Increased circulating sTIM-3 has been associated with progression and poorer therapeutic response in several malignancies, but the evidence remains heterogeneous and does not support standalone diagnostic or predictive use [85].

TIM-3 should therefore be interpreted as part of an immune context. Co-expression with PD-1, LAG-3, TIGIT, CTLA-4, or CEACAM1, the identity of expressing cells, tumor type, disease stage, and previous immunotherapy may all alter its meaning. Experimental strategies include monoclonal or bispecific antibodies, combined TIM-3 and PD-1 pathway blockade, and cell-based modification. These approaches aim to restore antitumor immunity or overcome resistance, but TIM-3-directed treatment remains investigational, and its expression is not yet a universally validated treatment-selection biomarker [84–86].

Within the proposed actionability framework, CD30 and CD38 may reach category A when they identify a validated therapeutic target in a defined malignancy. In other settings, they more often function as category B or investigational category D markers. TIM-3 currently belongs predominantly to category D. For all three markers, interpretation requires integration with histology, immunophenotype, disease stage, imaging, previous treatment, and, when relevant, reassessment at relapse.

### Nomenclature-sensitive and less standardized markers

Some tumor-associated biomarkers are difficult to interpret, not only because of limited specificity, but also because different names, biological forms, and assay targets are used across clinical and research settings. TATI/SPINK1/PSTI and tissue polypeptide antigen illustrate two forms of this problem. In the first, several names refer to the same protein but emphasize different biological contexts. In the second, the marker represents an assay-defined group of cytokeratin-derived antigens rather than one unique analyte.

SPINK1, TATI, and PSTI refer to the same serine protease inhibitor. SPINK1 is the gene and protein designation; PSTI reflects its physiological role as pancreatic secretory trypsin inhibitor; and TATI is commonly used for the circulating or tumor-associated protein in oncology. In addition to inhibiting trypsin, SPINK1 may act as an acute-phase reactant and promote proliferation, survival, and EGFR-associated signaling in selected tumor cells. Increased tissue expression or serum and urinary concentrations have been associated with aggressive behavior and adverse outcomes in several malignancies [87].

These associations do not make SPINK1 a general cancer-detection marker. Pancreatitis, biliary disease, inflammation, and impaired renal clearance may increase circulating concentrations independently of malignancy. Germline *SPINK1* variants, including N34S, must also be distinguished from tumor-associated overexpression: they are linked primarily to pancreatitis susceptibility and do not establish the presence of a SPINK1-positive tumor. Direct targeting of SPINK1 or downstream EGFR/MAPK signaling remains investigational. Its most plausible roles are risk stratification in selected tumor phenotypes and identification of biologically distinct subgroups for future targeted-treatment studies.

TPA is an assay-defined tissue polypeptide antigen related mainly to cytokeratins 8, 18, and 19, which occur widely in simple epithelia and many carcinomas [88, 89]. Tissue cytokeratin patterns can support the classification of poorly differentiated tumors or metastases of uncertain origin. Circulating TPA, however, consists of released or partially degraded cytokeratin-associated material and should not be equated directly with tissue immunohistochemistry.

Because cytokeratin release accompanies epithelial turnover, necrosis, and tissue injury, serum TPA is not organ-specific and may increase in malignant as well as non-malignant conditions. Earlier studies suggested potential value for assessing tumor burden, treatment response, or recurrence in selected carcinomas. In invasive bladder cancer, pretreatment concentrations were associated with disease stage and prognosis and showed possible follow-up value despite limited diagnostic sensitivity [90]. Differences between TPA, tissue polypeptide-specific antigen, and other cytokeratin assays further limit comparison across studies because antibody specificities and measured epitopes are not interchangeable.

TATI/SPINK1/PSTI and TPA, therefore, belong mainly to categories C or D. Their potential lies in selected prognostic, burden-related, or longitudinal applications. In contrast, inconsistent terminology, benign elevation, and assay dependence prevent universal diagnostic use. Reporting should specify the biological material, assay method, and molecular form measured rather than relying on the abbreviated marker name alone.

## Marker-specific evidence map: practical interpretation by biomarker group

### Group-based marker atlas

A marker-specific atlas is useful only if it shows where a biomarker is clinically strong and where it is likely to mislead. For this reason, the detailed characteristics of individual markers are summarized in Tables 2 and 3 rather than repeated in long descriptive subsections. Table 2 maps the principal cancer associations, screening and diagnostic value, prognostic relevance, monitoring value, recurrence or surveillance role, therapeutic-target relevance, and final actionability category. Table 3 focuses on the main interpretation traps: frequent false-positive conditions, false-negative or non-secretory states, and the clinical warning that should accompany the result. This format follows the practical logic of tumor-marker use more closely than a purely biochemical classification. The key issue is not whether a marker is biologically linked to cancer, but whether its result can support a defined decision in a defined clinical setting [21, 28].

To summarize this decision-oriented interpretation, Table 2 maps each marker according to its dominant clinical use and actionability level, while deliberately separating screening, diagnosis, prognosis, monitoring, recurrence surveillance, and therapeutic-target relevance, because evidence supporting one use cannot be automatically extrapolated to another [3, 4, 21, 28].

**Table 2:** Marker-specific clinical use and actionability matrix

Marker	Main cancer associations	Screening	Diagnosis	Prognosis	Monitoring	Recurrence/ surveillance	Therapeutic-target relevance	Dominant actionability range
CEA	Colorectal; gastrointestinal; selected lung and breast cancers	No	Limited adjunct	Yes	Yes, if baseline-positive	Established mainly in colorectal cancer follow-up	No	A/B
CA-125	Epithelial ovarian cancer; serosal involvement	Not for population screening	Adjunct in adnexal mass assessment	Yes	Established in ovarian cancer	Context-dependent	No	A/B
CA19-9	Pancreaticobiliary; gastrointestinal cancers	No	Adjunct only	Yes	Yes, if baseline-positive	Limited/context-dependent	No	B
CA-72-4	Gastric and mucinous gastrointestinal/ovarian cancers	No	Limited adjunct	Context-dependent	Context-dependent	Limited	No	B/C
CA15-3	Breast cancer, especially metastatic disease	No	No/limited	Yes	Useful in selected metastatic settings	Limited; not standalone; not recommended for routine surveillance after primary breast cancer therapy in asymptomatic patients.	No	B
CYFRA 21 − 1	Non-small-cell lung cancer; squamous malignancies	No	Adjunct only	Yes	Context-dependent	Limited	No	B/C
SCC antigen	Cervical, head and neck, lung, esophageal, and other squamous cancers	No	Adjunct only	Yes	Context-dependent	Context-dependent	No	B/C
HE4	Epithelial ovarian cancer; adnexal-mass risk assessment	No	Adjunct with clinical/imaging context	Yes	Context-dependent	Limited	No	B
PSA	Prostate cancer risk assessment and follow-up	Risk-adapted only	Adjunct; not cancer-specific	Yes	Yes	Yes, especially after treatment	No	A/B
AFP	Hepatocellular carcinoma; nonseminomatous germ-cell tumors	Risk-adapted in HCC only	Established in the germ-cell tumor context, adjunct in HCC	Yes	Yes	Yes, context-dependent	No	A/B
β-hCG	Germ-cell tumors; trophoblastic disease	No	Established in an appropriate clinical context	Yes	Yes	Yes	No	A
Calcitonin	Medullary thyroid carcinoma	Selected high-risk/nodule contexts	Strong niche use	Yes	Yes	Yes	No	A
Thyroglobulin	Differentiated thyroid carcinoma after thyroidectomy	No	Poor before thyroidectomy	Yes	Established after thyroidectomy	Established, if antibody status is considered	No	A
Chromogranin A	Well-differentiated neuroendocrine neoplasms	No	Adjunct only	Yes	Yes, if baseline-positive and confounders controlled	Context-dependent	No	B/C
5-HIAA	Serotonin-producing neuroendocrine tumors	No	Adjunct in the carcinoid syndrome context	Context-dependent	Yes, if the secretory phenotype is present	Context-dependent	No	B
NSE	Small-cell lung cancer; high-grade neuroendocrine tumors	No	Adjunct only	Yes	Context-dependent	Limited	No	B/C
S100/S100B	Melanoma; neural injury contexts	No	No/limited	Yes	Context-dependent in advanced melanoma	Context-dependent	No	B/C
LDH	Germ-cell tumors; lymphoma; melanoma; advanced solid cancers	No	No	Established in selected prognostic models	Burden-related only	Limited	No	C
TK1	Hematological malignancies; selected solid tumors	No	Investigational/limited	Context-dependent	Context-dependent	Investigational/limited	No	C/D
Ferritin	Hematological and advanced solid cancers; inflammatory/tumor-burden contexts	No	No	Context-dependent	Limited	No/limited	No	C
β2-microglobulin	Multiple myeloma; lymphoid malignancies	No	No	Established in selected hematological models	Context-dependent	Limited	No	C
CD30	Classical Hodgkin lymphoma; anaplastic large-cell lymphoma; selected lymphoid malignancies	No	Phenotypic adjunct	Yes	Context-dependent	Reassessment may be relevant after targeted therapy	Yes, validated in defined settings	A/B
CD38	Multiple myeloma; plasma-cell disorders; selected hematological malignancies	No	Phenotypic adjunct	Yes	Yes, in defined plasma-cell contexts	Context-dependent	Yes, validated in plasma-cell disorders	A/B
TIM-3	Immune-exhaustion and tumor–immune-context marker; selected hematological and solid tumors	No	No	Investigational/context-dependent	Investigational	Investigational	Investigational	D
TATI/SPINK1/PSTI	Pancreatic, gastrointestinal, ovarian, prostate, and selected other cancers	No	Limited/investigational	Context-dependent	Limited	Limited	Investigational	C/D
TPA	Epithelial cancers; cytokeratin-associated tumor burden	No	No/limited	Context-dependent	Context-dependent	Limited	No	C/D

Table note: Categories refer to marker–indication pairs rather than to biomarkers as fixed entities. A = established use in a defined clinical context; B = clinically useful adjunct, not standalone; C = mainly prognostic, burden-related, or longitudinal; D = investigational, poorly standardized, or highly context-dependent. Combined labels such as A/B or B/C indicate the range of clinically plausible uses across indications. “No” does not imply biological irrelevance; it means that the marker should not be used for that purpose in routine decision-making

Because most routine tumor biomarkers are conditional rather than disease-specific signals, Table 3 summarizes the main clinical false-positive and false-negative scenarios that should be considered before an abnormal or normal result is converted into a diagnostic or management decision [1, 3, 4, 35].

**Table 3:** False-positive and false-negative interpretation traps

Marker	Frequent false-positive conditions	Frequent false-negative / non-secretor scenarios	Key clinical warning
CEA	Smoking; liver disease; cholestasis; pancreatitis; inflammatory bowel disease; benign pulmonary disease	Early or low-volume disease; non-secretory tumors	A mild isolated rise is not recurrence until trend, liver status, smoking, and imaging context are checked.
CA-125	Menstruation; pregnancy; endometriosis; pelvic inflammatory disease; serosal inflammation; benign ovarian cysts	Early ovarian cancer: mucinous or non-secretory tumors	Interpret with menopausal status, ultrasound morphology, and inflammatory/serosal context.
CA19-9	Cholestasis; cholangitis; pancreatitis; benign biliary obstruction; liver disease	Lewis-negative phenotype; early disease; non-secretory tumors	Read with bilirubin and cholestatic enzymes; reassess after biliary drainage when appropriate.
CA-72-4	Benign gastrointestinal disease; inflammatory or mucinous conditions	Low-volume gastric/mucinous disease; non-secretory tumors	Not suitable for general gastric cancer screening; use only as an adjunctive signal.
CA15-3	Benign breast disease; liver disease; inflammatory conditions; some non-breast malignancies	Early breast cancer; marker-negative metastatic disease	Most useful only when elevated at baseline in established disease.
CYFRA 21 − 1	Benign lung disease; renal dysfunction; inflammatory epithelial injury	Low-volume disease; non-squamous or weakly shedding tumors	Supports assessment in selected cancers but does not identify the primary site.
SCC antigen	Benign squamous disease; renal impairment; inflammatory skin, lung, or gynecologic conditions	Low-volume disease; heterogeneous or poorly secreting tumors	Elevation reflects squamous-cell turnover, not necessarily malignant progression.
HE4	Renal impairment; older age; smoking; benign gynecologic or pulmonary disease	Early ovarian cancer: non-epithelial or weakly expressing tumors	Useful only with a clinical and imaging context; renal function is essential.
PSA	Benign prostatic hyperplasia; prostatitis; urinary retention; recent manipulation or instrumentation	Some clinically significant cancers: small-volume or low-PSA tumors	Prostate-associated, not prostate-cancer-specific; interpret with risk profile and prostate context.
AFP	Pregnancy; chronic hepatitis; cirrhosis; hepatic regeneration	AFP-negative HCC; pure seminoma; early germ-cell tumors	A normal AFP does not exclude HCC; true AFP elevation argues against pure seminoma.
β-hCG	Pregnancy; pituitary secretion; renal dysfunction; selected non-trophoblastic tumors	Marker-negative germ-cell tumors; early or treated disease	Always interpret with sex, age, pregnancy status, and tumor histology.
Calcitonin	C-cell hyperplasia; renal impairment; smoking; selected drugs or neuroendocrine conditions	Rare calcitonin-low or calcitonin-negative medullary thyroid carcinoma	Mild elevation requires confirmation and thyroid-specific context.
Thyroglobulin	Residual benign thyroid tissue; thyroiditis; incomplete thyroidectomy	Anti-thyroglobulin antibodies; dedifferentiated disease	Most meaningful after thyroidectomy; antibody status must accompany every result.
Chromogranin A	Proton pump inhibitors; atrophic gastritis; renal impairment; cardiovascular disease	Non-secretory or poorly differentiated neuroendocrine tumors	Medication and renal review are mandatory before imaging escalation.
5-HIAA	Serotonin-rich diet; interfering medication; incomplete dietary preparation	Non-serotonin-secreting tumors; incomplete urine collection; renal dysfunction	Useful mainly in the context of serotonin-producing tumors and carcinoid syndrome.
NSE	Benign neuroendocrine activation; neurological injury; systemic illness	Non-secretory tumors; weak neuroendocrine phenotype	Interpret only as an adjunct; unexpected rises require verification.
S100/S100B	Neurological injury; inflammation; renal dysfunction; non-malignant systemic conditions	Low-volume melanoma; non-expressing tumors	Mainly a burden/prognostic signal in known melanoma, not a melanoma-detection test.
LDH	Liver injury; muscle injury; infection; inflammation; hemolysis-related tissue injury	Low-volume or indolent disease	A blunt burden marker; never used to localize cancer.
TK1	Benign proliferation; infection; inflammation; hematopoietic activation	Slowly proliferating tumors; assay-dependent low signal	Best treated as a context-dependent proliferation marker.
Ferritin	Infection; inflammation; liver disease; iron overload; transfusion history; metabolic disease	Tumors without systemic inflammatory or iron-metabolism signal	Hyperferritinaemia is not evidence of cancer by itself.
β2-microglobulin	Renal impairment; inflammation; immune activation	Low-burden disease; interpretation masked by renal changes	Always interpret with renal function and inflammatory status.
CD30	Reactive immune activation; infection; inflammation; autoimmune disease	Sampling error; heterogeneous or treatment-suppressed expression	Confirm that the expression is on neoplastic cells, not only reactive background cells.
CD38	Normal plasma cells, activated immune cells, and reactive marrow changes	Antigen downregulation or masking after anti-CD38 therapy	Positivity is actionable only in the right cellular and disease context.
TIM-3	Immune activation; exhausted/reactive immune-cell populations	Compartment-specific expression missed by unsuitable sampling	An immune-context marker, not a standalone diagnostic or predictive test.
TATI/SPINK1/PSTI	Pancreatitis; biliary disease; inflammation; renal impairment	Tumors without SPINK1/TATI overexpression	Terminology and sample type must be clear before interpretation.
TPA	Benign epithelial turnover; tissue injury; inflammation	Low-shedding tumors; assay-dependent weak signal	A cytokeratin-turnover signal, not an organ-specific cancer marker.

Table note: Table 3 lists clinical and biological interpretation traps. Analytical and pre-analytical problems such as assay-platform change, heterophile antibodies, biotin interference, hook effect, sample handling, and formal reporting safeguards are addressed separately in Table 4

Classical circulating epithelial and glycoprotein markers, including CEA, CA-125, CA19-9, CA-72-4, CA15-3, CYFRA 21 − 1, SCC antigen, and HE4, form the largest and most familiar group. They are widely available and often useful, but their main weakness is equally clear: most are not sufficiently tumor-specific or organ-specific for population screening or standalone diagnosis. Their strongest value usually appears after cancer has already been diagnosed, especially when the marker was elevated at baseline and can be followed during treatment or surveillance. CEA is useful mainly in colorectal cancer follow-up, CA-125 in epithelial ovarian cancer monitoring, CA15-3 in selected metastatic breast cancer settings, and CA19-9 as an adjunct in pancreaticobiliary malignancy rather than as a general cancer test [1, 3, 4, 8]. The common error is to convert a nonspecific elevation into a diagnostic label before considering inflammation, cholestasis, smoking, renal function, liver disease, tumor phase, and previous marker behavior.

Organ-associated markers, such as PSA, AFP, β-hCG, calcitonin, and thyroglobulin, appear more specific, but even here the specificity is conditional. PSA is prostate-associated, not prostate-cancer-specific. AFP and β-hCG are highly informative in the management of germ-cell tumors, but both may be affected by physiological or nonmalignant conditions. Calcitonin is central to medullary thyroid carcinoma, while thyroglobulin becomes clinically powerful mainly after thyroidectomy, when normal thyroid tissue has been removed from the Eqs [50, 51, 54–57, 59, 60]. These examples show that clinical specificity is often achieved by the right setting: the same marker may be weak before diagnosis but highly informative once histology, treatment status, and disease phase are known.

Neuroendocrine and neural-lineage markers should be interpreted through tumor phenotype. Chromogranin A is meaningful only if the tumor has neuroendocrine secretory features and major confounders, especially proton pump inhibitor use and renal dysfunction, have been addressed. 5-HIAA is most relevant for serotonin-producing neuroendocrine tumors, particularly when carcinoid syndrome is suspected or established. NSE and S100B are mainly adjunctive markers in selected high-grade neuroendocrine tumors and melanoma [61–68]. In this group, a normal value may simply mean that the tumor does not secrete the analyte, while an elevated value may reflect medication, diet, renal impairment, hemolysis, or tissue injury. The first clinical question should therefore be biological: is this tumor expected to produce this marker?

LDH, TK1, ferritin, and β2-microglobulin have different roles. They usually do not point to a specific primary tumor. Instead, they reflect tumor burden, proliferation, tissue injury, inflammation, immune activation, or impaired clearance. LDH and β2-microglobulin are embedded in several prognostic models, particularly in hematological malignancies, but their values are easily distorted by hemolysis, liver or muscle injury, infection, inflammation and renal dysfunction [69–77]. TK1 and ferritin may provide useful information in selected contexts, but neither should be used as a general diagnostic marker. Their clinical value is strongest when the question is prognostic or longitudinal, not when the question is “where is the cancer?”

CD30, CD38, and TIM-3 should not be forced into the same interpretive model as serum tumor markers. CD30 and CD38 may identify actionable therapeutic targets in defined diseases, especially classical Hodgkin lymphoma, anaplastic large-cell lymphoma, multiple myeloma, and systemic light-chain amyloidosis. TIM-3 is more experimental and mainly reflects immune dysfunction, checkpoint biology, treatment resistance, and tumor–immune interaction [78–86]. For these markers, the relevant issues are not a serum threshold but the identity of the cell-expressing population, staining pattern, expression intensity, heterogeneity, prior therapy, and integration with morphology and immunophenotype. A positive result in reactive immune cells does not carry the same meaning as expression by neoplastic cells.

TATI/SPINK1/PSTI and TPA illustrate another problem: terminology itself can become clinically unsafe. SPINK1, TATI, and PSTI refer to the same protein but are used in different biological and clinical contexts. TPA, in contrast, is not a single unique molecular analyte but an assay-defined cytokeratin-associated marker, mainly related to cytokeratins 8, 18, and 19 [87–90]. In both cases, the abbreviated marker name is not enough. The report should make clear what was measured, in which biological material, by which assay, and for what clinical purpose. Without that precision, comparison across studies or clinical decisions may be misleading.

### Key clinical examples of misleading interpretation

The specificity trap becomes most visible in everyday clinical situations. The following examples are deliberately practical because they represent the kinds of results that may trigger unnecessary imaging, invasive work-up, or premature reassurance.

#### CA19-9 in cholestasis and benign biliary obstruction

CA19-9 is often associated with pancreaticobiliary cancer, but cholestasis is one of the classic situations in which this association becomes dangerous. Choledocholithiasis, cholangitis, cholecystitis, and benign biliary obstruction may produce substantial CA19-9 elevations. The mechanism is not mysterious: biliary epithelial irritation, impaired excretion, and raised intraductal pressure can all increase circulating CA19-9 without cancer. In jaundiced patients, benign and malignant conditions may overlap markedly. One clinical series showed that CA19-9 correlated with bilirubin in benign jaundice and decreased after biliary drainage in all benign cases [25, 91]. Therefore, CA19-9 measured during active obstruction should be read together with bilirubin, cholestatic enzymes, infection markers, and imaging. If the clinical situation allows, reassessment after biliary decompression is usually more informative than acting on the first value alone.

#### CA-125 in benign gynecologic, physiological, and inflammatory states

CA-125 is often treated as an ovarian cancer marker, but biologically, it is closer to a marker of coelomic epithelial irritation. Endometriosis, menstruation, pregnancy, pelvic inflammatory disease, benign ovarian cysts, uterine fibroids, and inflammatory conditions involving the peritoneum, pleura, or pericardium may all increase CA-125 [3, 92]. The same numerical value, therefore, has a different meaning in different women. A postmenopausal woman with a complex adnexal mass and a rising CA-125 is not equivalent to a menstruating premenopausal woman with endometriosis. Age, menopausal status, menstrual timing, pregnancy, ultrasound morphology, and signs of serosal inflammation are not secondary details; they define the interpretation. CA-125 remains valuable in established epithelial ovarian cancer, but an isolated elevation should not be treated as a diagnosis. Importantly, CA-125 also illustrates that biochemical detectability is not identical to clinical actionability. In the MRC OV05/EORTC 55,955 randomized trial, early treatment of ovarian cancer relapse triggered by rising CA-125 alone did not improve overall survival compared with delayed treatment based on clinical or symptomatic relapse [93]. Thus, a rising CA-125 concentration may correctly anticipate relapse, but earlier detection is clinically valuable only when it leads to an intervention that improves meaningful patient outcomes.

#### CEA in smokers and liver disease

CEA is useful in colorectal cancer surveillance, but mild elevation is common outside malignant recurrence. Active smoking may raise the baseline, and liver disease or cholestasis may impair clearance. Pancreatitis, inflammatory bowel disease, and some pulmonary disorders can also increase CEA [1, 45, 94]. In practice, a small isolated rise should first prompt a check of smoking status, liver tests, inflammatory activity, and the previous CEA trajectory. In a patient after colorectal cancer treatment, a sustained rise measured by the same assay deserves attention, but even then, it should lead to clinically appropriate confirmation, not automatic restaging based on the marker alone.

#### PSA in benign prostatic hyperplasia and prostatitis

PSA is produced by benign and malignant prostate epithelium. Benign prostatic hyperplasia, prostatitis, urinary retention, recent instrumentation, and prostate manipulation may all elevate PSA [50, 51]. This is why PSA is best understood as a risk-modifying marker, not a cancer-confirming marker. Interpretation should include age, previous PSA values, prostate volume, PSA density, urinary symptoms, signs of infection, digital rectal examination, and, where indicated, multiparametric magnetic resonance imaging. When a reversible inflammatory or procedural cause is likely, repeat testing after clinical stabilization may avoid unnecessary biopsy, provided that the patient’s overall risk does not require immediate investigation.

#### Chromogranin A during proton pump inhibitor treatment

Chromogranin A is a frequent source of false alarms. Proton pump inhibitors induce hypochlorhydria and secondary hypergastrinaemia, which stimulate enterochromaffin-like cells and may substantially increase chromogranin A without neuroendocrine tumor progression [36, 61, 62]. Renal impairment and atrophic gastritis add further complexity. A high chromogranin A value without medication review is therefore incomplete information. When feasible and clinically safe, repeat measurement after supervised interruption or substitution of proton pump inhibitor therapy may prevent unnecessary imaging. The marker is most helpful when it is clearly elevated at baseline, confounders are controlled, and the trend agrees with symptoms and radiology.

#### NSE elevation caused by hemolysis

NSE is vulnerable to a simple but important pre-analytical problem: erythrocytes and platelets contain enolase. Hemolysis can therefore produce a falsely high serum NSE and simulate neuroendocrine tumor activity or disease progression [37, 64]. Limited hemolysis may not be obvious visually, so the hemolysis index and specimen-handling information matter. An unexpected rise in NSE should usually be repeated with careful sampling before it is interpreted oncologically. Correcting or commenting on hemolysis at the laboratory level is useful, but prevention and recognition of the pre-analytical error remain more important.

#### β2-microglobulin in renal impairment

β2-microglobulin is released during cellular turnover and immune activation, but it is cleared predominantly by the kidneys. Reduced glomerular filtration may therefore raise serum β2-microglobulin independently of tumor burden [35, 75–77]. This is especially relevant in multiple myeloma and lymphoid malignancies, where renal impairment may be part of the disease, a comorbidity, or a treatment-related complication. A high value may thus reflect both tumor biology and renal function. Interpretation should always include creatinine, estimated glomerular filtration rate, inflammatory status, and renal trajectory. Serum β2-microglobulin should also not be confused with tissue-level *B2M* gene alterations, which represent a distinct molecular mechanism associated with immune escape.

#### LDH in tissue injury, hemolysis, and inflammation

LDH is a useful but blunt marker. It is present in almost all tissues and rises with cell injury, necrosis, and high turnover. Tumor burden, hypoxia, and aggressive disease may increase LDH, but so may hemolysis, liver injury, skeletal-muscle damage, infection, and systemic inflammation [69]. A hemolyzed sample alone can create a misleading elevation. LDH is therefore most defensible as a prognostic or burden-related marker within a known disease model. It should not be used to identify the primary tumor or to change treatment without concordant clinical, laboratory, or imaging evidence.

These examples all lead to the same practical rule: discordant tumor-marker results should be checked before they are believed. The safest interpretation is usually the one that asks whether the result fits the patient, disease phase, sample quality, assay, and previous trajectory. The most clinically instructive examples of misleading tumor-marker interpretation are summarized in Box 1, highlighting common confounding contexts and the practical checks required before diagnostic or therapeutic escalation.

**Figure:**
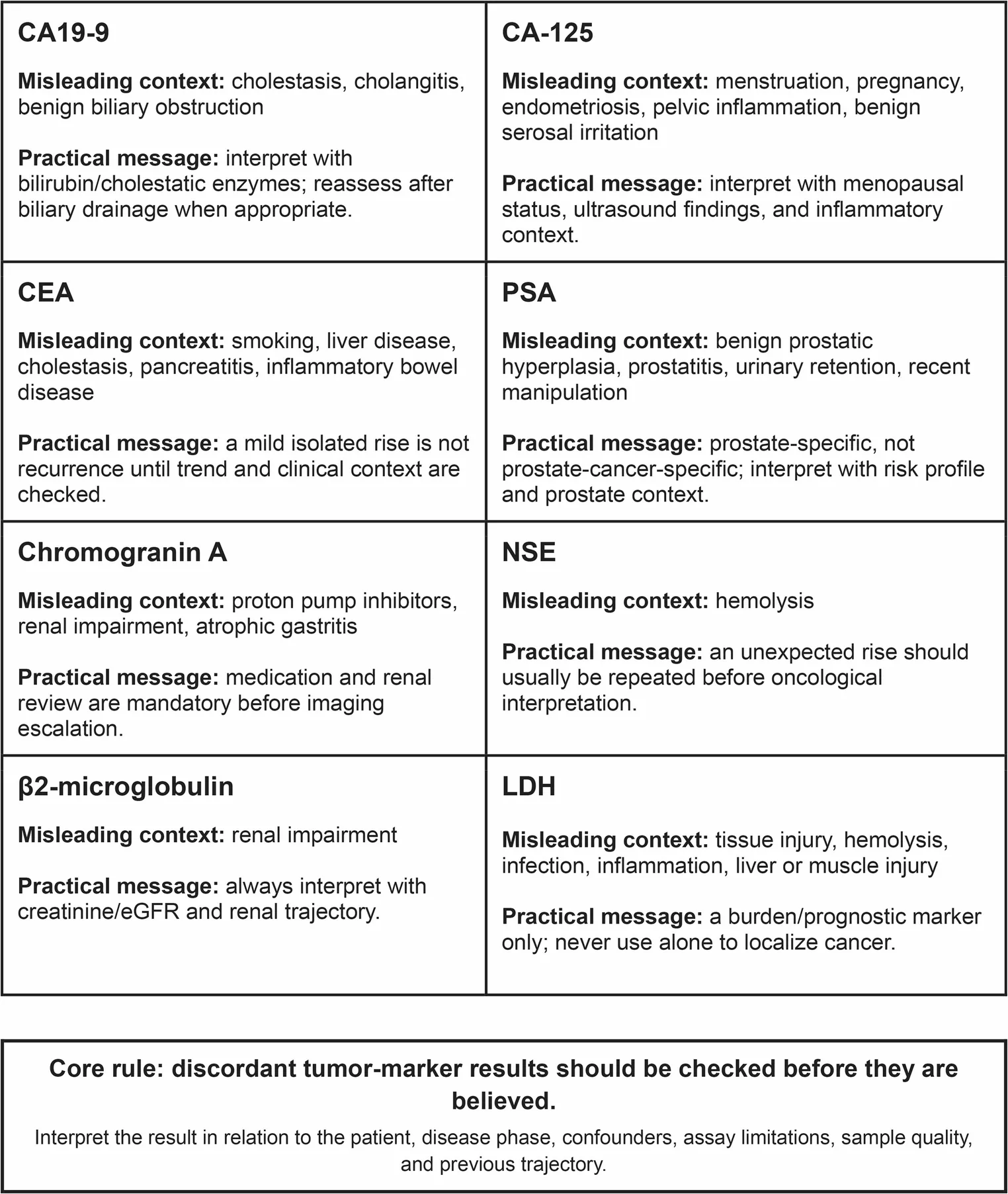
Box 1. Key clinical examples of misleading interpretation Selected routine tumor-biomarker situations in which isolated results may mislead clinical decision-making

### Clinical actionability categories

For clinical use, it is more helpful to classify marker applications than to classify markers in isolation. The same analyte may be strongly actionable in one setting and weak or misleading in another. The proposed categories, therefore, apply to a marker–indication pair, not permanently to the biomarker itself. This approach is consistent with the principle that analytical validity, clinical validity, and clinical utility must be considered separately before a biomarker result is used to guide management [21, 28].

#### Category A: established, guideline-supported use in a defined context

Category A application has a defined patient population, validated indication, accepted analytical method, and a result that can reasonably guide management. Examples include AFP and β-hCG in germ-cell tumors, thyroglobulin after treatment of differentiated thyroid carcinoma, calcitonin in medullary thyroid carcinoma, CEA in colorectal cancer surveillance, CA-125 in monitoring epithelial ovarian cancer, and CD30 or CD38 when they identify eligibility for approved targeted therapy. Category A does not mean universal usefulness. It means that the marker is useful for a specific decision in a specific clinical context [4, 21].

#### Category B: clinically useful adjunct, but not standalone

Category B markers contribute to assessment but should not determine management alone. CA19-9 in pancreaticobiliary disease, CA15-3 in metastatic breast cancer, HE4 in adnexal-mass evaluation, and CYFRA 21 − 1, SCC antigen, NSE, or S100B in selected tumors often belong here. These markers may support imaging, histology, clinical examination, or risk models, but action should depend on concordant evidence. Their main risk is overinterpretation when the biomarker is treated as stronger than the clinical context allows [3, 4].

#### Category C: mainly prognostic, burden-related, or longitudinal marker

Category C includes markers that usually describe burden, proliferation, injury, inflammation, or prognosis rather than a specific diagnosis. LDH, TK1, ferritin, β2-microglobulin, and TPA often fall into this category. They may be valuable in defined prognostic systems or when serial changes parallel disease behavior, but they rarely identify the origin of malignancy or select a specific treatment [69, 70, 75, 88]. β2-microglobulin in multiple myeloma illustrates the nuance: it has strong prognostic relevance in the right framework, but outside that framework, it remains nonspecific and renal-function dependent.

#### Category D: investigational, poorly standardized, or highly context-dependent use

Category D does not mean biologically unimportant. It means that clinical validity, analytical standardization, or clinical utility is not yet strong enough for routine decision-making. TIM-3 as a treatment-selection biomarker, therapeutic targeting of SPINK1, several TK1 applications, and soluble immune checkpoint markers currently fall mainly into this category. These markers may be important for research, patient stratification, or future trials, but routine use should remain cautious until assays, thresholds, and marker-guided interventions are better validated [84, 87].

This category system is meant to keep interpretation honest. It prevents weak markers from being promoted into diagnostic tests, but it also prevents useful markers from being dismissed simply because they are not universally cancer-specific. The practical meaning of categories A–D is summarized in Box 2, which distinguishes established, adjunctive, prognostic or longitudinal, and investigational marker–indication uses. In a predictive, preventive, and personalized framework, the question is not only whether the value is abnormal. The more relevant question is whether this marker is valid for this patient, in this disease phase, using this assay, and whether the result can change a clinical decision.

**Figure:**
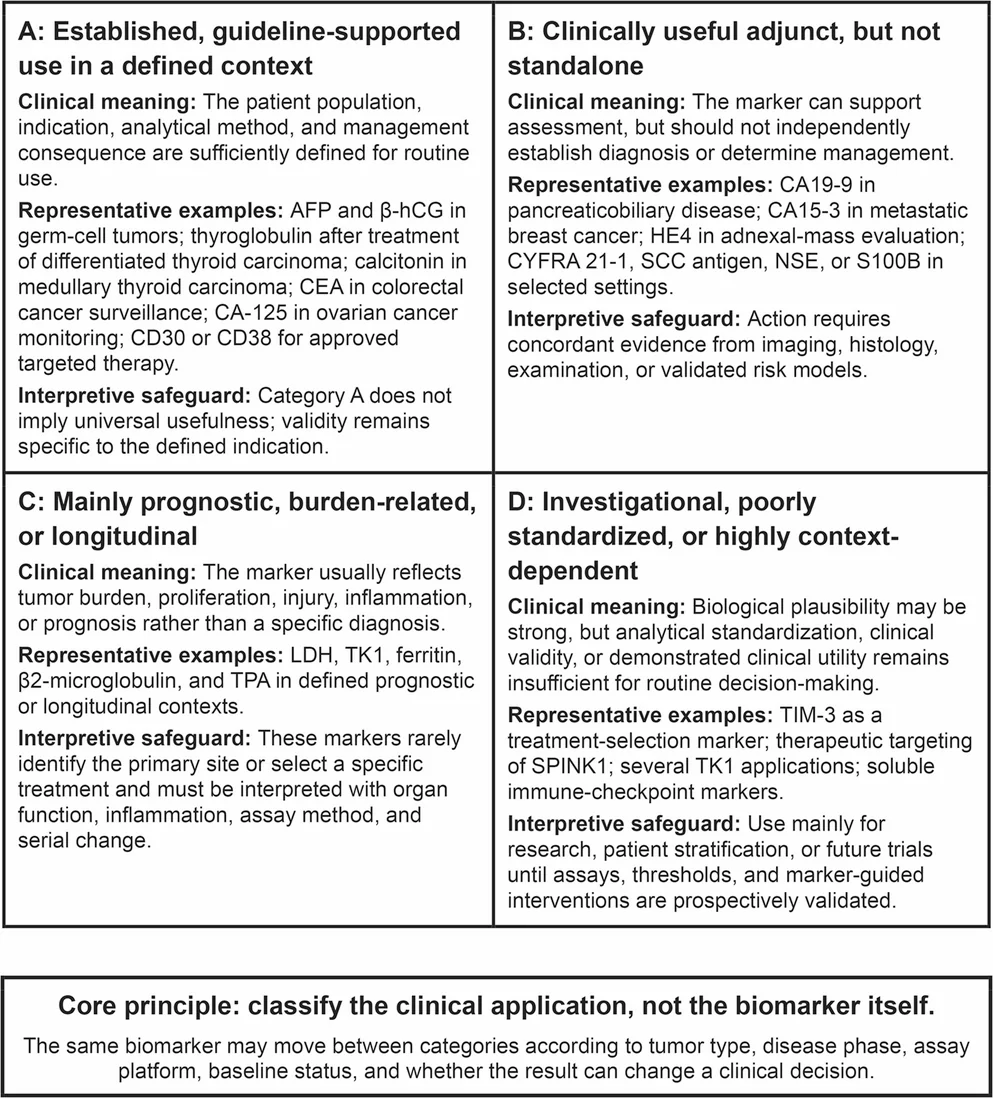
Box 2. Clinical actionability categories. Categories apply to marker–indication pairs, not to biomarkers as fixed entities

## From laboratory result to clinical decision: a 3PM-oriented algorithm

A clinically useful tumor-marker algorithm should begin before the result is interpreted. The first question is not whether the value is abnormal, but whether the marker was ordered to answer a question that it can reasonably answer. This distinction is essential because analytical validity, clinical validity, and clinical utility are not interchangeable properties. A technically robust assay may still be clinically weak if it is used in the wrong tumor type, at the wrong disease phase, or without a management consequence [21, 28]. The conceptual mini-scheme includes three levels of biomarker evidence (Box 3):

**Figure:**
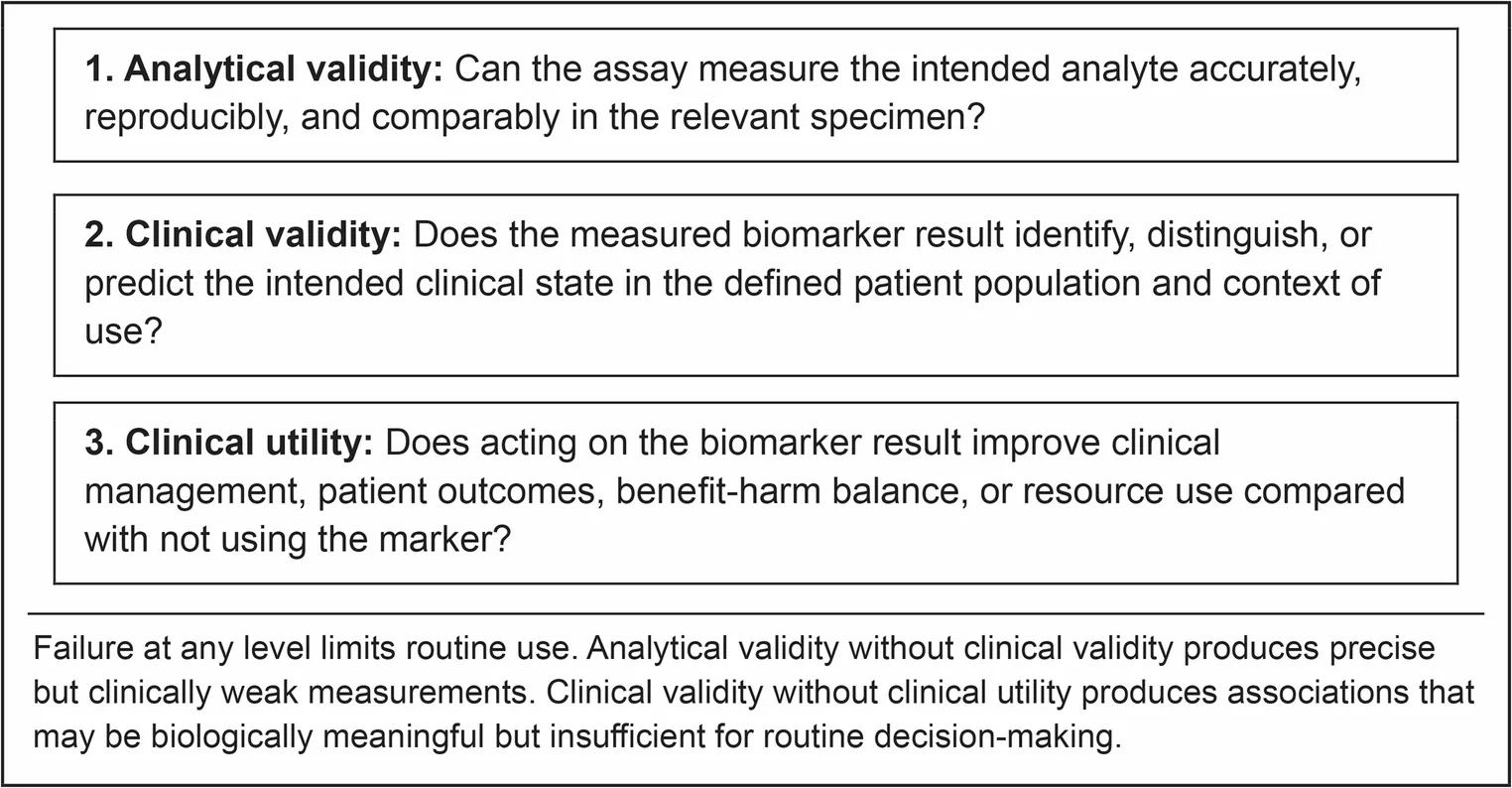
Box 3. Three levels of the biomarker evidence

**Fig. 2:**
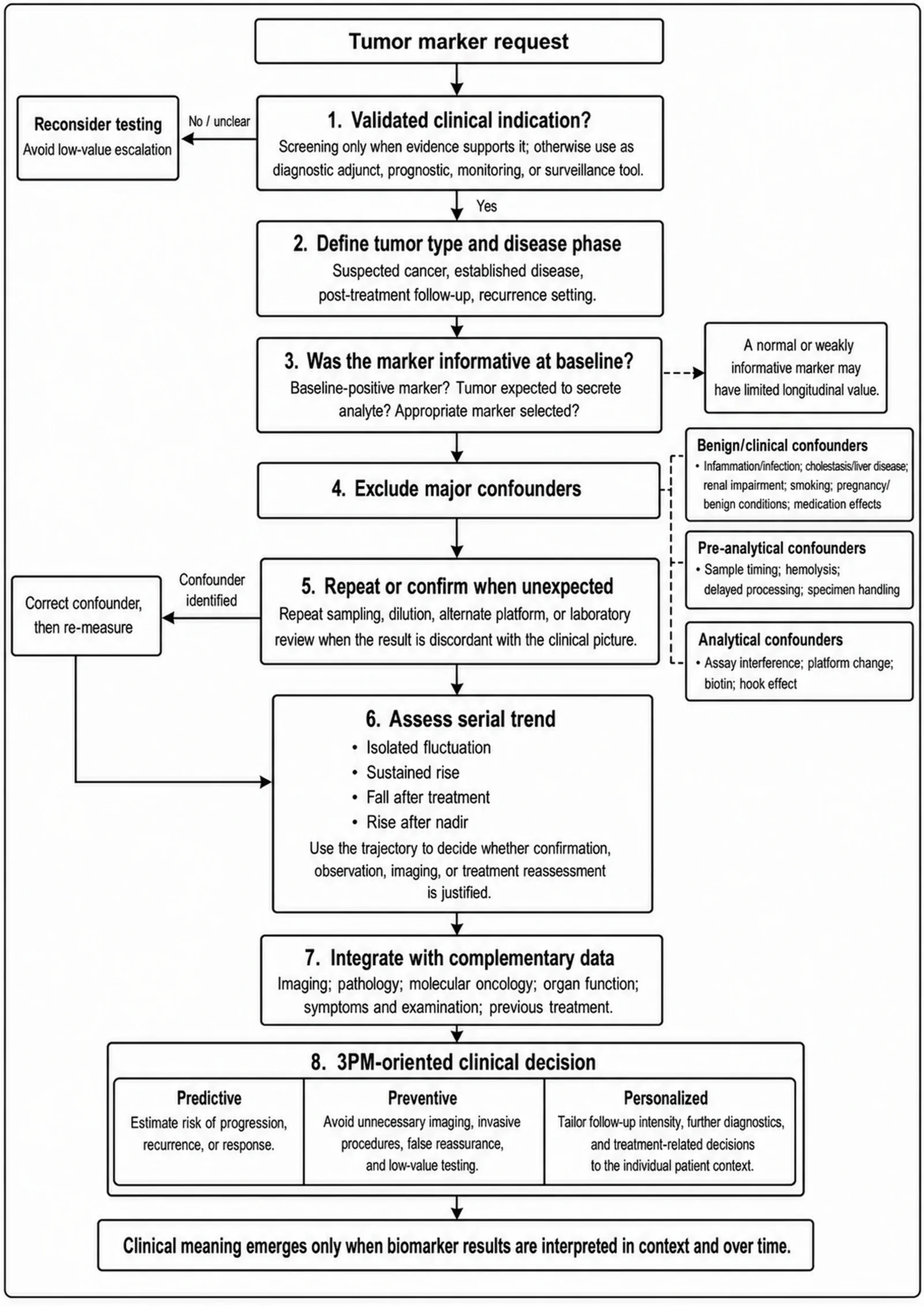
3PM-oriented clinical decision workflow for routine tumor-marker interpretation The figure presents a stepwise clinical workflow for interpreting routine tumor biomarkers within a predictive, preventive, and personalized medicine framework. The process begins with verification of a validated clinical indication, because biomarker testing should be avoided when the expected result cannot support a defined diagnostic, prognostic, monitoring, surveillance, or treatment-related decision. Interpretation then requires definition of the tumor type and disease phase, assessment of whether the marker was informative at baseline, and systematic exclusion of benign clinical, pre-analytical, and analytical confounders. Unexpected or discordant results should prompt repeat sampling, dilution studies, testing on an alternative assay platform, or laboratory review before diagnostic escalation. Serial trends should then be used to decide whether confirmation, observation, imaging, or treatment reassessment is justified. The final interpretation should integrate biomarker dynamics with imaging, pathology, molecular oncology, organ function, symptoms, examination findings, and prior treatment. This approach supports predictive assessment of progression, recurrence, or response; prevents unnecessary imaging, invasive procedures, false reassurance, and low-value testing; and enables personalized decisions adapted to the individual patient context.

In routine practice, the safest interpretation is therefore not threshold-centered, but indication-centered and utility-aware. This stepwise logic is summarized in Fig. 2.

### Five-question checklist and green/amber/red interpretation zones

Before a tumor marker result is used to guide clinical action, five questions should be answered. First, was the marker requested for a validated or at least defensible indication? Second, is the marker appropriate for the suspected or known tumor type and for the current disease phase? Third, was the marker elevated at baseline (before treatment) or at the time of established disease? Fourth, are any benign pre-analytical or analytical confounders present? Fifth, would the result change imaging, biopsy, treatment, surveillance intensity, or follow-up timing? If the answer to the fifth question is no, the result may still be biologically interesting, but it should not drive management.

The result can then be assigned to one of three practical interpretation zones. The green zone describes a marker measured in a validated context, elevated or informative at baseline, followed on the same analytical platform, and showing a clinically concordant serial trend. In this setting, the marker may support assessment of response, residual disease, relapse suspicion, or disease burden, particularly when the change exceeds expected analytical and biological variation [19, 20]. This logic is consistent with the current 3PM shift from delayed reactive decision-making toward earlier risk recognition, targeted prevention, and personalized intervention [7].

The amber zone is the most common and dangerous in everyday practice. It includes a mild isolated elevation, a borderline rise, a result obtained during infection, cholestasis, renal impairment, recent procedure, pregnancy, medication exposure, hemolysis, or a change measured after switching assay platforms. In this situation, escalation should usually pause. The more rational step is to repeat measurement under controlled conditions, preferably in the same laboratory, together with review of liver and renal function, inflammatory indices, sample quality, medication history, and possible immunoassay interference [33, 42]. A single amber result should not be used to assign a cancer label.

The red zone applies when a marker shows a sustained or marked rise in a high-risk clinical context and the trajectory is concordant with symptoms, imaging, endoscopy, pathology, or molecular evidence. This zone justifies diagnostic acceleration: targeted imaging, biopsy, multidisciplinary review, closer surveillance, or treatment-response reassessment may be appropriate. However, red does not mean autonomous. A tumor marker should rarely be the sole reason for starting, stopping, or changing systemic treatment, unless that use is explicitly supported for the marker–indication pair. The algorithm should therefore reduce both forms of error: delayed action in genuinely high-risk trajectories and overreaction to analytically or biologically explainable noise.

In this way, the algorithm is not only a diagnostic safeguard but also a preventive strategy against low-value testing, avoidable imaging, unnecessary invasive procedures, and delayed recognition of truly high-risk trajectories.

### Integration with imaging, pathology, molecular oncology, and personalized baseline

Routine tumor markers should be interpreted as one layer of evidence, not as a parallel diagnostic system. Imaging defines anatomical extent, pathology confirms tissue identity, immunohistochemistry clarifies lineage and therapeutic phenotype, and molecular oncology identifies actionable genomic or pathway-level vulnerabilities. Circulating protein markers can complement these domains, but they cannot substitute for them. Their contribution is strongest when they provide a longitudinal signal that is biologically plausible, reproducible, and aligned with the patient’s disease course.

The personalized baseline is central to this interpretation. A marker that was never elevated at diagnosis is usually weak for surveillance, even if it is traditionally associated with that tumor type. Conversely, a marker that was clearly elevated before treatment and then falls to a nadir after effective therapy may become useful for follow-up, provided that assay consistency and confounders are controlled for. In this sense, the patient’s prior values serve as an internal reference interval. A small rise within population limits may be relevant in one patient, whereas a stable mild elevation may be uninformative in another [19, 30]. Recent EPMA J evidence from non-invasive metabolomic monitoring in breast cancer also supports the broader 3PM principle that patient-specific longitudinal profiles may provide clinically relevant information beyond static group-level thresholds [95].

Discordance should trigger verification, not automatic escalation. If the marker rises while imaging and clinical status remain stable, the first step is to check timing, sample quality, assay platform, organ function, inflammation, and interference. If imaging suggests progression but the marker remains normal, the clinician should consider non-secretory disease, low marker expression, histological transformation, treatment-induced phenotype change, or simply the wrong marker for that tumor biology. A normal result should not be used to overrule a credible clinical or radiological signal.

This approach translates the 3PM concept into practical laboratory medicine. The predictive component lies in using validated baseline-dependent kinetics to refine the probability of response, recurrence, or progression. The preventive component lies in avoiding low-value testing, unnecessary imaging, invasive procedures, and anxiety caused by nonspecific abnormalities. The personalized component lies in anchoring interpretation to the individual patient: tumor phenotype, disease phase, baseline secretion, organ function, treatment history, assay method, and trajectory over time [7]. In treatment-selection contexts, the same logic applies to immune and molecular biomarkers: marker-guided decisions require standardized assays, clinically meaningful thresholds, and prospective validation before they can be used as reliable instruments for personalized oncology [96]. The final decision should therefore emerge from the convergence of biomarker dynamics and the broader clinical picture, not from an isolated numerical value.

Its practical value will increase when routine marker kinetics are integrated with imaging, pathology, molecular profiling, organ function data, and patient-specific longitudinal records, rather than being interpreted as isolated laboratory events.

## Implementation, future directions, and conclusions

The proposed framework is intended to be used at the point where most interpretation errors begin: after a numerical result has been released, but before it is converted into a clinical decision. Implementation, therefore, requires more than clinician education. It requires a reporting culture in which tumor marker results are delivered with sufficient analytical and clinical context to prevent overinterpretation. A minimally useful report should include the assay method or platform, reference interval, previous result, absolute and percentage changes, date of the previous measurement, and a brief interpretive warning when major confounders are likely. For markers commonly affected by renal dysfunction, cholestasis, hemolysis, smoking, pregnancy, medication exposure, or immunoassay interference, the report should explicitly remind the clinician that the value is not disease-specific. During follow-up, the same laboratory and analytical platform should be used whenever possible, because method-dependent differences may mimic clinically meaningful marker kinetics [33].

To translate these principles into laboratory practice, Table 4 summarizes the main analytical, pre-analytical, and reporting safeguards that should accompany routine tumor-marker interpretation, particularly when serial monitoring, assay comparability, or unexpected discordance may affect clinical decisions [20, 33, 37–42].

**Table 4:** Analytical, pre-analytical, and reporting safeguards for routine tumor-marker interpretation

Issue	Affected markers/examples	Clinical consequence	Recommended reporting/interpretation note
Assay-platform variability	Especially CA15-3, CA19-9, CA-125; also relevant to many immunoassay-based markers	Apparent rise or fall may reflect method change rather than biology.	Report assay method/platform and advise same-laboratory, same-platform follow-up whenever possible.
Missing previous value	All serially monitored markers	The current result may be overread as abnormal or underread as stable.	Display the previous value, date, absolute change, and percentage change in the report.
Biological and analytical variation	CA-125, CEA, CA19-9, AFP and other serial markers	Small changes may be within expected variation.	Interpret changes against baseline, nadir, trend, and expected reference change, not only the population reference interval.
Hemolysis	NSE; LDH	False elevation may simulate neuroendocrine activity, tissue injury, or progression.	Report hemolysis index where relevant and recommend repeat sampling if the result is discordant.
Delayed or inconsistent sample handling	NSE; peptide/protein markers; markers requiring defined collection conditions	Degradation, cellular release, or handling artifact may distort the value.	Standardize sample type, processing time, storage, and collection timing for longitudinal monitoring.
Heterophile or human anti-animal antibodies	CA19-9 and other immunoassay-based markers	False high or false low result; possible unnecessary imaging or invasive work-up.	Suspect interference when the result is clinically implausible; consider dilution, blocking reagents, or alternative platform testing.
Biotin interference	Immunoassays using biotin–streptavidin systems; possible relevance to several tumor markers	Direction of error depends on assay format; false high or false low results may occur.	Ask about high-dose biotin supplements and repeat after appropriate withholding when clinically feasible.
High-dose hook effect	PSA, β-hCG, and other high-concentration immunometric assays	Very high analyte concentration may appear falsely low or negative.	Suspect when the measured value is incompatible with tumor burden; request serial dilution.
Renal-function dependence	β2-microglobulin; HE4; chromogranin A; SCC antigen	Reduced clearance may mimic tumor burden or progression.	Interpret together with creatinine/eGFR and renal trajectory.
Liver dysfunction or cholestasis	CA19-9, CEA, AFP, and selected epithelial markers	Impaired clearance, inflammation, or biliary obstruction may mimic malignancy or progression.	Interpret with bilirubin, cholestatic enzymes and liver injury markers; reassess after clinical stabilization when appropriate.
Medication-related distortion	Chromogranin A with proton pump inhibitors: selected assay-specific drug effects	Drug effect may be mistaken for tumor activity.	Include medication review in interpretation; repeat after supervised withdrawal/substitution when safe and relevant.
Discordance with clinical picture	Any marker	Risk of forcing the result into an incorrect disease narrative.	Do not act on an isolated discordant value before checking indication, sample quality, assay method, confounders, and repeat/confirmatory testing.

Table note: Table 4 focuses on analytical, pre-analytical, and reporting safeguards. Clinical false-positive and false-negative scenarios are summarized separately in Table 3; marker-specific clinical use and actionability are summarized in Table 2

Taken together, these clinical and analytical safeguards identify several situations in which routine tumor-marker testing is more likely to generate misleading escalation than clinically useful information; these “do-not-order” or “do-not-act” scenarios are summarized in Box 4.

**Table:** Box 4. Situations in which routine tumor-marker testing should usually be avoided or deferred

• Asymptomatic population screening without a validated indication • Broad tumor-marker panels without a clinical question • Marker not elevated at baseline and used for surveillance • Isolated mild elevation during infection, cholestasis, renal deterioration, or pregnancy • Result obtained after assay-platform change • Hemolyzed or otherwise compromised sample • No realistic management consequence	

Laboratory reporting should also move from isolated values toward longitudinal interpretation. A mildly abnormal but stable result may have less clinical importance than a smaller but reproducible rise from the patient’s own nadir. Conversely, a single elevated value obtained during infection, biliary obstruction, renal deterioration, or after an assay change should not trigger diagnostic escalation before the result is verified. The practical reporting unit should therefore include not only the current concentration but also the trajectory: baseline value, nadir, direction and magnitude of change, and concordance with the expected clinical course. This is consistent with the view that biomarker monitoring requires specific evaluation of disease-status discrimination, timing, and the clinical consequences of acting on a marker change [20].

For clinicians, implementation can be reduced to a simple rule: no tumor-marker result should generally be acted on in isolation until its indication, baseline informativeness, confounders, assay consistency, and management consequence have been checked. This rule does not weaken the clinical value of tumor markers. On the contrary, it protects their value by using them where they are informative and withholding action where they are only nonspecific biochemical noise. In predictive, preventive, and personalized medicine, the aim is not to order more markers, but to extract better decisions from markers that are already used. The potential preventive value of this approach is clinically relevant because it may reduce low-value testing, avoidable imaging, unnecessary biopsies, and patient anxiety caused by clinically weak abnormalities.

Future research should therefore prioritize clinical utility rather than biomarker enthusiasm. New or repurposed tumor-marker applications should pass through analytical validation, clinical validation, demonstration of clinical value, and regulatory or guideline-level assessment before they are promoted into routine use [21]. This is particularly important for markers currently placed in category C or D, where biological plausibility is often stronger than evidence for management benefit. Prospective studies should test whether marker-guided decisions improve outcomes that matter to patients: earlier actionable relapse detection, better treatment allocation, reduced diagnostic burden, fewer false alarms, better quality of life, or improved cost-effectiveness. Earlier, biochemical detection alone was not enough.

The next step will be multimodal interpretation. Serial tumor marker kinetics should be integrated with imaging, pathology, immunohistochemistry, molecular profiling, organ function data, symptoms, treatment timing, and patient-specific longitudinal records. This direction is aligned with current EPMA thinking, where digital and molecular biomarkers are not viewed as isolated measurements, but as components of an interpretable, patient-centred risk model [7]. Recent EPMA J evidence also supports the broader principle that longitudinal, non-invasive profiling may capture patient-specific biological information beyond static thresholds [95]. For routine tumor markers, this means that the future does not lie in using CEA, CA-125, CA19-9, PSA, LDH, or β2-microglobulin as standalone cancer signals. Their future lies in calibrated integration.

AI-supported models may help, but only if they solve a clinical problem rather than decorate the workflow. Useful models should combine serial marker kinetics with relevant clinical and analytical variables, flag discordant results, identify high-risk trajectories, and recommend verification when confounding is probable. They should also remain transparent enough for clinical use. A black-box model that amplifies assay artifacts, ignores platform changes, or treats all abnormal values as equivalent would simply automate the specificity trap. In oncology, 3PM-guided stratification is strongest when predictive biomarkers, treatment context, resistance mechanisms, and cost-effective personalized decisions are evaluated together rather than separately [96].

Several practical research priorities follow from this framework. First, marker-specific reference change values should be refined for clinically relevant populations and assay platforms. Second, prospective trials should define when a change in a marker should prompt repeat testing, imaging, biopsy, treatment modification, or observation. Third, laboratory information systems should be redesigned to display previous values, assay method, confounder alerts, and interpretive comments in a clinically usable format. Fourth, multimodal algorithms should be externally validated across laboratories, tumor types, disease stages, and healthcare systems. Fifth, future guidelines should more explicitly distinguish among screening, diagnosis, prognosis, monitoring, recurrence surveillance, therapeutic targeting, and investigational use. These roles should not be merged under the vague label of “tumor marker”.

### Clinical and conceptual limitations

Several clinical and conceptual limitations of this framework should be acknowledged. First, the proposed algorithm is not intended to replace disease-specific guidelines, histopathological confirmation, radiological assessment, or validated molecular decision pathways. It is a decision-support structure for routine tumor-marker interpretation, not an autonomous diagnostic rule. Second, the actionability categories used in this review are pragmatic marker–indication categories rather than a formally validated scoring system. They are intended to discipline interpretation, not to create a new regulatory or guideline classification.

Third, the framework deliberately focuses on selected routine, widely accessible, and interpretation-prone tumor biomarkers. It does not provide a complete catalog of all serum, tissue-based, genomic, liquid-biopsy, imaging, or composite biomarker tools. Therefore, the absence of a marker from the tables should not be interpreted as evidence of low biological or clinical importance. Instead, it reflects the scope of this review and the decision to prioritize markers that are frequently encountered in routine practice and commonly misinterpreted as cancer-specific signals.

Fourth, clinical utility remains context-dependent. A biomarker may be analytically robust and clinically associated with a malignancy, yet still fail to improve patient management if the result does not change imaging, biopsy, surveillance intensity, treatment selection, or patient-relevant outcomes. This limitation is especially important for markers with mainly prognostic, burden-related, investigational, or assay-dependent applications. The proposed framework should therefore be updated as assay standardization, disease-specific guidelines, molecular diagnostics, liquid-biopsy technologies, and evidence from prospective marker-guided studies evolve.

### Conclusions and recommendations in the 3PM-framework

The clinical value of tumor biomarkers lies less in isolated abnormal values than in context-corrected longitudinal interpretation. Routine tumor biomarkers are most clinically useful when interpreted as conditional, dynamic, and imperfect clinical signals within a predictive, preventive, and personalized framework. Used in this way, they can support earlier recognition of meaningful risk, prevent unnecessary diagnostic escalation, and personalize follow-up according to tumor biology, disease phase, comorbidities, analytical context, and the individual patient’s trajectory. The central message is therefore practical: tumor-marker results should not be read as stand-alone evidence for or against cancer, but as decision-support signals that require clinical, laboratory, imaging, and pathological correlation.

Finally, in primary care for protecting individuals against health-to-disease transition as well as for individualized rehabilitation programs in secondary care, a 3PM-guided holistic approach is strongly recommended utilizing multi-level diagnostics. This approach is based on comprehensive individualized patient profiles including phenotyping and biomarker panels essentially reflecting systemic effects. To this end, a non-invasive tear fluid test utilizing mitochondria as vital biosensors and AI-based multi-professional data interpretation has been established, followed by the cost-effective targeted disease prevention and individualized rehabilitation programs in primary and secondary care respectively [7, 97–103].

## Data Availability

No datasets were generated or analysed during the current study.

## Abbreviations

3PM: predictive, preventive, and personalized medicine
AFP: alpha-fetoprotein
AI: artificial intelligence
ALK: anaplastic lymphoma kinase
B2M: beta-2-microglobulin gene
CA-125: cancer antigen 125
CA19-9: carbohydrate antigen 19-9
CA-72-4: cancer antigen 72-4
CA15-3: cancer antigen 15-3
CD: cluster of differentiation
CD30: cluster of differentiation 30
CD38: cluster of differentiation 38
CD366: cluster of differentiation 366
CEA: carcinoembryonic antigen
CEACAM1: carcinoembryonic antigen-related cell adhesion molecule 1
CTLA-4: cytotoxic T-lymphocyte-associated protein 4
CYFRA 21-1: cytokeratin fragment 21-1
DNA: deoxyribonucleic acid
EGFR: epidermal growth factor receptor
EPMA: European Association for Predictive, Preventive and Personalized Medicine
HAVCR2: hepatitis A virus cellular receptor 2
HCC: hepatocellular carcinoma
HE4: human epididymis protein 4
5-HIAA: 5-hydroxyindoleacetic acid
LAG-3: lymphocyte activation gene 3
LDH: lactate dehydrogenase
MAPK: mitogen-activated protein kinase
MEDLINE: Medical Literature Analysis and Retrieval System Online
N34S: asparagine-to-serine substitution at codon 34
NSE: neuron-specific enolase
PD-1: programmed cell death protein 1
PSA: prostate-specific antigen
PSTI: pancreatic secretory trypsin inhibitor
SANRA: Scale for the Assessment of Narrative Review Articles
SCC: squamous cell carcinoma
SPINK1: serine peptidase inhibitor Kazal type 1
S100B: S100 calcium-binding protein B
TATI: tumor-associated trypsin inhibitor
TIGIT: T-cell immunoreceptor with immunoglobulin and ITIM domains
TIM-3: T-cell immunoglobulin and mucin-domain containing protein 3
TK1: thymidine kinase 1
TNFRSF8: tumor necrosis factor receptor superfamily member 8
TPA: tissue polypeptide antigen
β-hCG: beta-human chorionic gonadotropin
β2-microglobulin: beta-2-microglobulin
